# Connections between Exoproteome Heterogeneity and Virulence in the Oral Pathogen Aggregatibacter actinomycetemcomitans

**DOI:** 10.1128/msystems.00254-22

**Published:** 2022-06-13

**Authors:** Yanyan Fu, Sandra Maaβ, Marines du Teil Espina, Anouk H. G. Wolters, Yanan Gong, Anne de Jong, Erwin Raangs, Girbe Buist, Johanna Westra, Dörte Becher, Jan Maarten van Dijl

**Affiliations:** a University of Groningen, University Medical Center Groningen, Department of Medical Microbiology, Groningen, the Netherlands; b University of Greifswald, Institute of Microbiology, Department of Microbial Proteomics, Greifswald, Germany; c Department of Biomedical Sciences of Cells and Systems, University of Groningen, University Medical Center Groningen, Groningen, the Netherlands; d Chinese Center for Disease Control and Prevention, National Institute for Communicable Disease Control and Prevention, Collaborative Innovation Center for Diagnosis and Treatment of Infectious Diseases, Beijing, China; e University of Groningen, Groningen Biomolecular Sciences and Biotechnology Institute, Department of Molecular Genetics, Groningen, the Netherlands; f University of Groningen, University Medical Center Groningen, Department of Rheumatology and Clinical Immunology, Groningen, the Netherlands; Leiden University

**Keywords:** *Aggregatibacter actinomycetemcomitans*, exoproteome, serotype, virulence, secreted proteins, virulence factors

## Abstract

Aggregatibacter actinomycetemcomitans is a Gram-negative bacterial pathogen associated with severe periodontitis and nonoral diseases. Clinical isolates of A. actinomycetemcomitans display a rough (R) colony phenotype with strong adherent properties. Upon prolonged culturing, nonadherent strains with a smooth (S) colony phenotype emerge. To date, most virulence studies on A. actinomycetemcomitans have been performed with S strains of A. actinomycetemcomitans, whereas the virulence of clinical R isolates has received relatively little attention. Since the extracellular proteome is the main bacterial reservoir of virulence factors, the present study was aimed at a comparative analysis of this subproteome fraction for a collection of R isolates and derivative S strains, in order to link particular proteins to the virulence of A. actinomycetemcomitans with serotype b. To assess the bacterial virulence, we applied different infection models based on larvae of the greater wax moth Galleria mellonella, a human salivary gland-derived epithelial cell line, and freshly isolated neutrophils from healthy human volunteers. A total number of 351 extracellular A. actinomycetemcomitans proteins was identified by mass spectrometry, with the S strains consistently showing more extracellular proteins than their parental R isolates. A total of 50 known extracellular virulence factors was identified, of which 15 were expressed by all investigated bacteria. Importantly, the comparison of differences in exoproteome composition and virulence highlights critical roles of 10 extracellular proteins in the different infection models. Together, our findings provide novel clues for understanding the virulence of A. actinomycetemcomitans and for development of potential preventive or therapeutic avenues to neutralize this important oral pathogen.

**IMPORTANCE** Periodontitis is one of the most common inflammatory diseases worldwide, causing high morbidity and decreasing the quality of life of millions of people. The bacterial pathogen Aggregatibacter actinomycetemcomitans is strongly associated with aggressive forms of periodontitis. Moreover, it has been implicated in serious nonoral infections, including endocarditis and brain abscesses. Therefore, it is important to investigate how A. actinomycetemcomitans can cause disease. In the present study, we applied a mass spectrometry approach to make an inventory of the virulence factors secreted by different clinical A. actinomycetemcomitans isolates and derivative strains that emerged upon culturing. We subsequently correlated the secreted virulence factors to the pathogenicity of the investigated bacteria in different infection models. The results show that a limited number of extracellular virulence factors of A. actinomycetemcomitans have central roles in pathogenesis, indicating that they could be druggable targets to prevent or treat oral disease.

## INTRODUCTION

Periodontitis is one of the most common inflammatory diseases worldwide. This disease impacts heavily on people’s quality of life, as it will inevitably lead to tooth loss if left untreated. The Gram-negative, nonmotile, facultative anaerobic bacterial pathogen Aggregatibacter actinomycetemcomitans is strongly associated with aggressive (grade C) periodontitis in young individuals and adolescents (1–3), and it is one of the most prevalent microorganisms in patients with periodontitis (4). Importantly, A. actinomycetemcomitans has been associated with serious nonoral infections, such as endocarditis and brain abscesses (5, 6).

When cultured *in vitro*, A. actinomycetemcomitans isolates display two phenotypes. Usually, colonies of fresh clinical isolates of A. actinomycetemcomitans have a rough (“R”), star-shaped, transparent appearance, and the bacteria adhere tightly to agar or the glassware and plastics used for liquid culture (6–10). However, after several passages, the colonies become smooth (“S”), star-negative, and opaque, and the bacteria no longer adhere tightly to surfaces (7–11). Of note, not only the adhesive properties of A. actinomycetemcomitans isolates can be related to the R and S colony phenotypes but also the production of various virulence factors (7, 12–14). For instance, it has been reported that R isolates produce more lipopolysaccharides and proteases than S strains (7). On the other hand, it has been proposed that S strains secrete leukotoxin to higher levels (15). Also, the S strains have been reported to invade epithelial cells more readily than the R isolates (16).

The isolates of A. actinomycetemcomitans have been classified into seven different serotypes (designated a to g) based on differences in the O-polysaccharide (O-PS) component of their lipopolysaccharides (LPS) (17–20). Variations in the structure and immunogenicity of O-PS of A. actinomycetemcomitans were shown to be relevant determinants for periodontal health or disease (21). In particular, the serotype a isolates were associated with healthy carriage or chronic periodontitis, while serotype b isolates were associated with aggressive periodontitis and tooth loss (21–24). An association with healthy carriage or oral disease is apparently less clear for serotype c isolates. Together, the prevalence and association of particular A. actinomycetemcomitans serotypes with periodontitis seem to vary depending on geography and ethnicity, but serotype b isolates appear to be most pathogenic. Accordingly, serotype b isolates were shown to produce the highest amounts of the leukotoxin LtxA, which is often regarded as the main virulence factor of A. actinomycetemcomitans (25, 26).

Since the genotype impacts the pathogenic potential of A. actinomycetemcomitans (2, 24, 27), an alternative approach for typing A. actinomycetemcomitans isolates is based on the *ltxA* promoter. Especially the JP2 genotype in serotype b isolates, which relates to a deletion in the *ltxA* promoter region, shows high leukotoxicity and is strongly linked to aggressive periodontitis (25, 26). Such JP2 isolates are most frequently encountered in North and West Africa, but their detection has also been reported for Caucasians living in other parts of the world (28, 29). In contrast, non-JP2 isolates that lack the deletion in the promoter region are more frequently detected in other geographical regions such as the European countries (30, 31). Furthermore, a subgroup of the non-JP2 serotype b isolates of A. actinomycetemcomitans, which display high leukotoxicity and a similar disease phenotype as JP2 isolates, can be identified based on the presence of the *cagE* gene for a putative exotoxin (32). Besides LtxA, A. actinomycetemcomitans also produces other virulence factors, including the cytolethal-distending toxin (CDT) (33), the peptidoglycan-associated lipoprotein (PAL) (34), and OmpA (35), but these are usually not taken into account for the distinction of A. actinomycetemcomitans isolates (17–20, 25, 26).

The bacterial exoproteome is generally considered the main reservoir of bacterial virulence factors (36, 37). In previous studies, we have shown that different exoproteome features of lineages of the major human pathogen Staphylococcus aureus can be related to this bacterium’s virulence and epidemic behavior (38, 39). Interestingly, we observed an extreme exoproteome heterogeneity among particular S. aureus lineages that are genetically closely related. To date, very little has been known about the exoproteome of A. actinomycetemcomitans and its possible variations. Therefore, the present study was aimed at a comparative analysis of the exoproteomes of different isolates of A. actinomycetemcomitans by liquid chromatography coupled to mass spectrometry (LC-MS/MS). In particular, we focused our study on five serotype b clinical isolates of A. actinomycetemcomitans with the R colony phenotype, because these are considered to be most pathogenic. Furthermore, to better understand the distinctive features of serotype b isolates with the R and S phenotypes, we also investigated the exoproteomes of derivative strains with the S phenotype, as well as the A. actinomycetemcomitans reference strain ATCC 29522, which also displays the S phenotype. Lastly, to correlate the exoproteome features of the different A. actinomycetemcomitans isolates to virulence, we applied infection models based on larvae of the greater wax moth Galleria mellonella, as well as a human salivary gland-derived epithelial cell line and freshly isolated neutrophils from healthy human volunteers.

## RESULTS

### Selection of A. actinomycetemcomitans with serotype b.

To select A. actinomycetemcomitans with serotype b, a total of 27 R isolates from periodontitis patients were screened by PCR, using the reference strains 14R (serotype a), ATCC 29522 and 29R (serotype b), and 1R (serotype c) as controls. Seven of these R isolates with serotype b were thus identified (see Fig. S1A in the supplemental material), all of which were subsequently classified by PCR as non-JP2 strains (Fig. S1B).

10.1128/msystems.00254-22.1 FIG S1 Selection of A. actinomycetemcomitans bacteria with serotype b. (A) PCR products diagnostic for serotype b A. actinomycetemcomitans obtained by multiplex PCR with primers P11, P12, P13, and P14 (Table S4) separated on a 1% agarose gel. (B) PCR products diagnostic for non-JP2 A. actinomycetemcomitans obtained by PCR with primers *ltxA* and *ltxB* (Table S4) separated on a 1% agarose gel. (C) Transformation rates of different A. actinomycetemcomitans isolates from the R to S phenotype. (D) LDS-PAGE analysis of the extracellular proteins of the investigated A. actinomycetemcomitans bacteria. The different lanes are labeled with the names of the respective R isolates or derivative S strains. The gel was stained by SimplyBlue staining. The protein band marked with an arrow probably represents LtxA, as judged by its mobility on LDS-PAGE. Molecular weight markers (M) are indicated in kilodaltons. Download FIG S1, PDF file, 1.0 MB. Copyright © 2022 Fu et al. 2022 Fu et al. https://creativecommons.org/licenses/by/4.0/ This content is distributed under the terms of the Creative Commons Attribution 4.0 International license.

To obtain derivative strains with the S phenotype, the seven R serotype b isolates were passaged through brain heart infusion (BHI) broth until homogeneous cultures were obtained. Subsequently, the S phenotype was verified by plating on BHI agar. Interestingly, different R isolates displayed different rates of transformation to the S phenotype (Fig. S1C). For instance, for the 4R isolate, 14 passages were necessary to obtain the S phenotype, while only 6 passages were required for the 23R isolate.

To assess further phenotypic differences between the different R isolates and the derivative S strains, lithium dodecyl sulfate (LDS)-PAGE was performed with secreted proteins that were collected from the culture medium. As shown in Fig. S1D, substantial differences were detectable in the respective protein patterns upon SimplyBlue staining of the gels. Based on these patterns, five R isolates with the most different extracellular protein patterns and the respective derivative S strains were selected for further analyses, along with the ATCC 29522 type strain that also displays the S phenotype.

### LtxA secretion.

**FIG 1:**
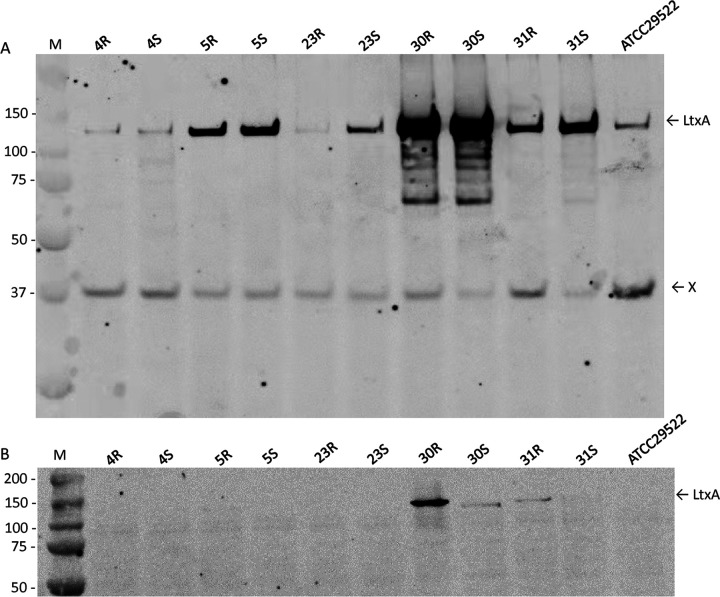
Western blotting of LtxA secretion. A. actinomycetemcomitans R isolates, their respective derivative S strains, and the reference strain ATCC 29522 were cultured in BHI broth. Upon 40 h of growth, secreted proteins were collected from the growth medium by TCA precipitation. (A) Equal amounts of collected extracellular protein were separated by LDS-PAGE and analyzed by immunoblotting with an LtxA-specific monoclonal antibody. Note that the band marked “X” relates to a protein that is recognized by the secondary goat anti-mouse antibody used to visualize the binding of the LtxA-specific antibody. (B) Cells of the different A. actinomycetemcomitans isolates were disrupted by bead beating, and equal amounts of cellular proteins were separated by LDS-PAGE and immunoblotting with LtxA-specific monoclonal antibodies as described for panel A. The masses of molecular weight markers (M) are indicated in kilodaltons to the left of the blots.

Since LtxA is regarded as the main virulence factor of A. actinomycetemcomitans, we assessed the secretion of this leukotoxin by Western blotting with a monoclonal LtxA-specific antibody (40). As shown in Fig. 1A, the isolates 5R, 30R, and 31R, as well as the respective derivative strains 5S, 30S, and 31S, secrete high amounts of LtxA into the growth medium. Inspection of the different cells by Western blotting showed that relatively low amounts of cell-associated LtxA were detectable in the cells of the 30R and 31R isolates (Fig. 1B). Interestingly, cells of the 30S strain also retained some LtxA but much less than the parental 30R isolate, showing that the phenotypic conversion from the R to the S phenotype was associated with differences in the secretion of at least one protein (i.e., LtxA). A similar effect was observed for the 31R and 31S pair, where the retention of LtxA was severely reduced in the derivative 31S strain. To investigate whether these differences might be associated with the presence or absence of the *cagE* marker gene for highly leukotoxic serotype b strains (32), their genome sequences were inspected. Interestingly, all strains were shown to contain the *cagE* gene, and hence, the observed differences in LtxA production by the here-investigated strains cannot be associated with the presence or absence of *cagE*.

### SNPs in derivative A. actinomycetemcomitans strains with the S phenotype.

**FIG 2:**
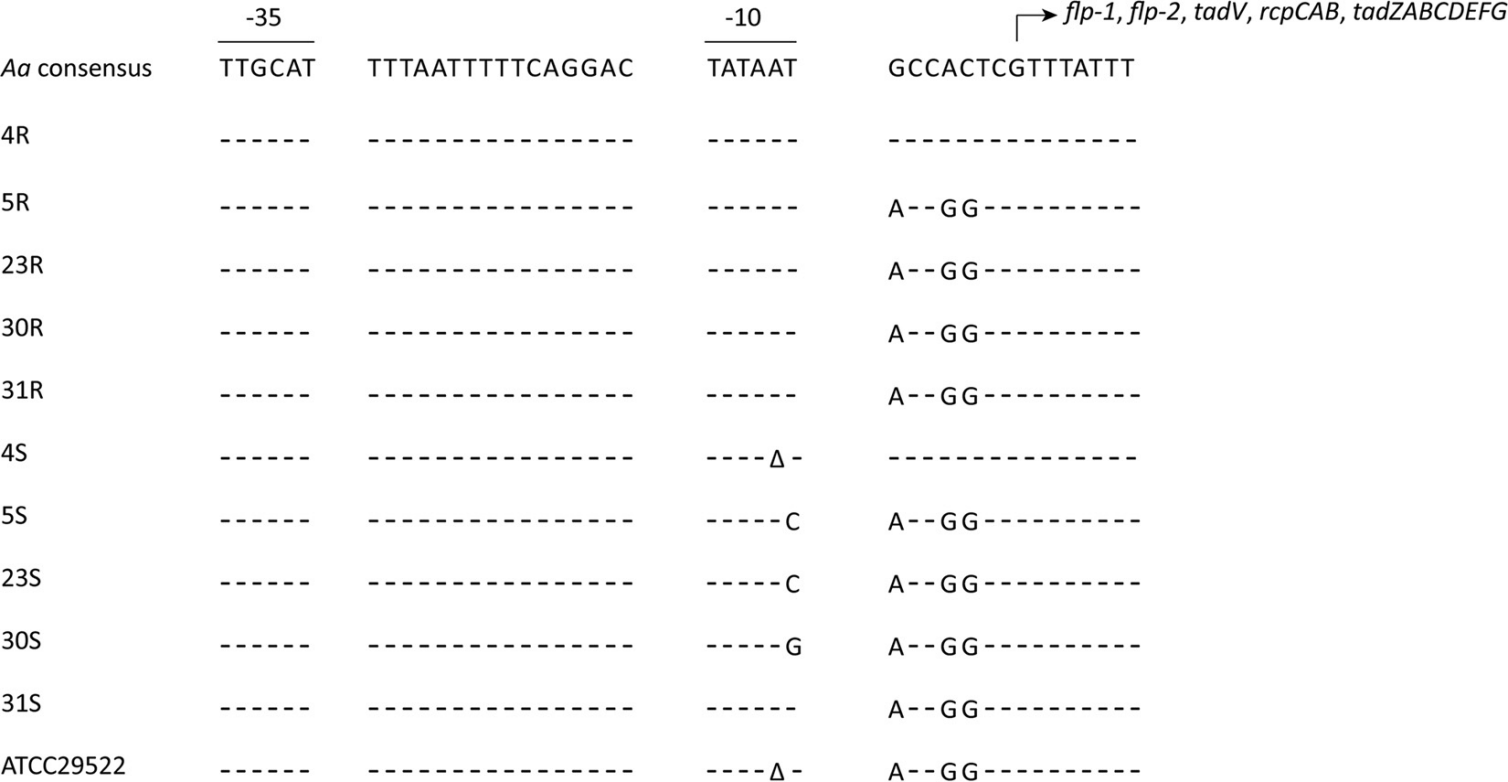
Localization of SNPs in the promoter regions of the investigated derivative A. actinomycetemcomitans (*Aa*) strains with the S colony phenotype. The consensus promoter region was previously defined by Wang et al. (41). Please note that with the exception of 4R/4S, the sequences of all other presently investigated R/S pairs and the ATCC type strain of A. actinomycetemcomitans deviate from the published promoter region with respect to 3 nucleotides downstream of the −10 region.

To pinpoint the mutations responsible for the change from the R to the S phenotype in the different investigated S strains of A. actinomycetemcomitans, we assessed the presence of single nucleotide polymorphisms (SNPs) using the MiSeq sequence data of the respective genomes. This revealed that in all S strains, except 31S, mutations had occurred in the previously defined −10 region of the *flp* promoter (Fig. 2; Table S1) (41). This implies that the expression of the *flp* operons in the 4S, 5S, 23S, and 30S strains is impaired, and it would explain the respective R-to-S conversion, since it was previously shown that fimbriae are important for the R phenotype of A. actinomycetemcomitans strains (9). In the case of the 31S strain, we noticed a SNP in the *recJ* gene, but further inspection of the genome sequence of this strain revealed that it lacks a large segment of the *flp-tad* locus. In particular, 10 genes ranging from *flp-1* to *tadC* were missing, with only the *tadDEFG* genes remaining in the 31S genome. Importantly, this genomic region was still present in all other sequenced S strains, including the ATCC type strain, which also has the S phenotype. Interestingly, by comparing the sequence data of the 4R, 5R, 23R, and 30R isolates with those of the ATCC type strain, we noticed the presence of the same SNP in the −10 region of the *flp* promoter in the ATCC strain as was detected in the 4S strain. This suggests that this mutation is responsible for the S phenotype of the ATCC strain.

10.1128/msystems.00254-22.4 TABLE S1 Genetic variations in different A. actinomycetemcomitans pairsTable S1, PDF file, 0.05 MB. Copyright © 2022 Fu et al. 2022 Fu et al. https://creativecommons.org/licenses/by/4.0/ This content is distributed under the terms of the Creative Commons Attribution 4.0 International license.

### TEM.

**FIG 3:**
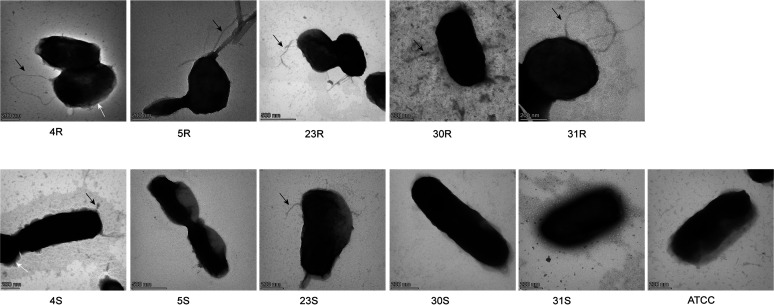
Transmission electron micrographs of A. actinomycetemcomitans fimbriae visualized by negative staining. A. actinomycetemcomitans bacteria with the R and S phenotypes were grown in BHI broth for 40 h. The bacteria were then harvested by centrifugation, and the bacterial pellets were washed with PBS. After staining, the different A. actinomycetemcomitans bacteria were imaged by TEM. Magnification is indicated by size bars.

To examine the presence of fimbriae on the cell surface of the investigated R and S pairs of A. actinomycetemcomitans, we applied transmission electron microscopy (TEM) with negative staining. As shown in Fig. 3, the different A. actinomycetemcomitans bacteria displayed different levels of fimbriation, with the R isolates displaying more abundant and longer fimbriae than the corresponding S strains. In particular, the 5R isolate showed bundles of fimbriae, whereas these were absent from the derivative 5S strain. Moreover, some R isolates showed short extensions associated with the outer membrane, as is most clearly evident for the 4R isolate in Fig. 3. Notably, some S strains, such as 4S and 23S, showed residual fimbriae. However, these fimbriae were shorter and more slender than those of their parental 4R and 23R isolates. As expected, no fimbriae were detectable for the 31S strain that lacks a large part of the *flp-tad* locus. Also, the ATCC type strain was completely devoid of fimbriae, which must be attributed to unidentified mutations in addition to the presently identified SNP in the *flp* promoter region of this strain.

### Distinction of rough A. actinomycetemcomitans isolates and derivative smooth strains based on extracellular proteins.

Since secreted proteins are considered the main reservoir of virulence factors produced by bacterial pathogens (36, 37), we examined the full complement of secretory proteins of the five selected R isolates, their derivative S strains, and the ATCC 29522 type strain. To this end, we applied an advanced proteomics approach based on LC-MS/MS. This allowed a quantitative analysis of the proteomic variations of A. actinomycetemcomitans strains with the serotype b.

**FIG 4:**
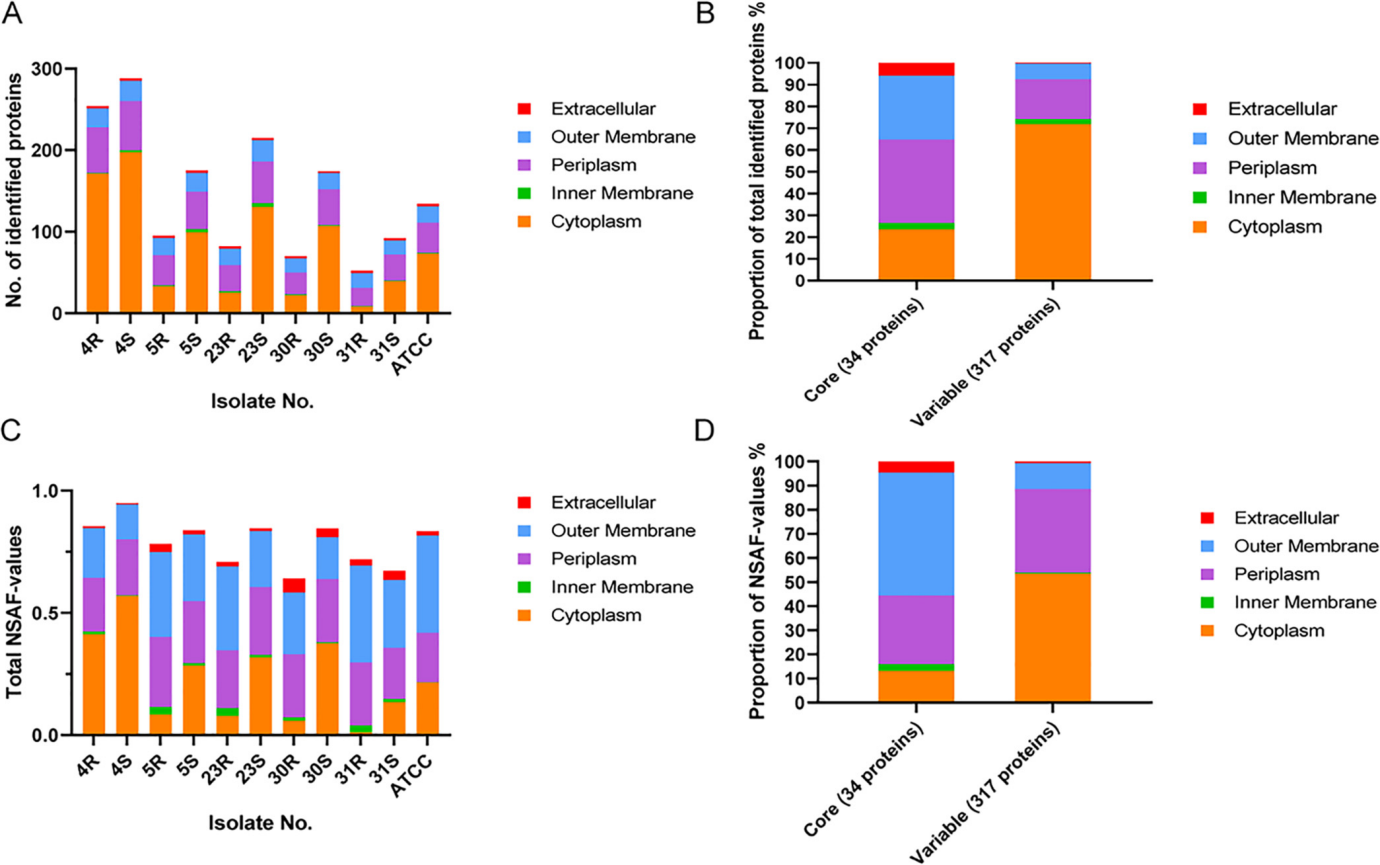
Subcellular localization prediction of extracellular A. actinomycetemcomitans proteins identified by proteomics. In total, 351 extracellular proteins were identified by proteomics. Their predicted subcellular localization was assessed using different bioinformatics tools and literature data. (A) Numbers of extracellular A. actinomycetemcomitans proteins and their predicted subcellular localization for each investigated R isolate and the respective S strain. (B) Relative numbers of core and variable extracellular A. actinomycetemcomitans proteins with particular predicted subcellular localizations. (C) Abundance based on normalized spectral abundance factor (NSAF) values and their predicted localization for each investigated R isolate and the respective S strain. (D) Relative abundance of core and variable proteins with particular predicted subcellular localizations.

A total of 351 proteins were identified in the combined exoproteome samples. With different bioinformatics tools and a manual curation based on literature, the predicted subcellular localization of all identified proteins was assessed. Interestingly, about two-thirds of the identified extracellular proteins, 236 (67.24%), represent typical cytoplasmic proteins. Furthermore, 71 proteins (20.23%) were predicted to have a periplasmic localization, eight proteins (2.28%) were predicted to be located in the inner membrane, and 33 proteins (9.4%) were predicted to be located in the outer membrane. Only three proteins were predicted to have an extracellular localization. All identified proteins and their respective abundance values are listed in Table S2. Interestingly, as shown in Fig. 4A, the number of identified proteins differs largely for the clinical R isolates and their respective derivatives with the S phenotype. In particular, the S strains always produce more extracellular proteins than their parental R isolates. This phenomenon is particularly evident for the 23R-23S and 30R-30S pairs, and it can be attributed mostly to variations in the numbers of identified extracellular cytoplasmic proteins (ECPs). Particularly, substantially higher numbers of ECPs were observed for S strains, which ranged between 197 (4S) and 39 (31S). In contrast, the numbers of ECPs observed for the R isolates ranged from 171 (4R) to 8 (31R), with the number of ECPs detected for the 4R isolate being relatively high (Fig. 4A). Conversely, relatively few differences between the R isolates and their S derivatives were observed in the numbers of predicted extracytoplasmic proteins. Together, these data show that a major difference between R isolates and their S derivatives concerns the numbers of detectable ECPs. In addition, we also investigated the amounts of proteins assigned to different locations based on the measured normalized spectral abundance factor (NSAF) values (Fig. 4C). Interestingly, the NSAF values indicate that in terms of protein amounts, the ECPs are relatively less abundant than suggested by the number of protein identifications, whereas the abundance of periplasmic and outer membrane proteins is relatively higher. Also, from the perspective of protein abundance, the 31R-31S pair is exceptional, since the 31R isolate excretes barely any ECPs, while the amount of ECPs excreted by the 31S isolate is relatively low compared to the other investigated S strains.

10.1128/msystems.00254-22.5 TABLE S2 All extracellular proteins of A. actinomycetemcomitans bacteria as identified by mass spectrometry and their respective abundance values. Download Table S2, XLSX file, 0.2 MB. Copyright © 2022 Fu et al. 2022 Fu et al. https://creativecommons.org/licenses/by/4.0/ This content is distributed under the terms of the Creative Commons Attribution 4.0 International license.

Among the 351 identified extracellular proteins, 34 proteins were detected for all five R isolates, the five S strains, and the type strain. The highest proportion of core extracellular proteins has a predicted periplasmic or outer membrane localization (41.18% or 29.41%, respectively) (Fig. 4B). Only one inner membrane protein was detected among the core exoproteome proteins. Importantly, our results show that two of the three proteins with a predicted extracellular localization belong to the core exoproteome, namely, LtxA and CdtB. Thus, in total, 317 proteins can be considered to represent the variable exoproteome of A. actinomycetemcomitans strains with serotype b, and ECPs represent the most abundant variable protein group (71.92%). Furthermore, the variable exoproteome includes 58 predicted periplasmic proteins (14.29%), seven inner membrane proteins, and one predicted extracellular protein (i.e., CdtA). The relative representation of ECPs within the core and variable exoproteomes in terms of protein abundance was also calculated. As shown in Fig. 4D, the relative ECP abundance was also low compared to the number of ECP identifications, especially in the core exoproteome. In contrast, the contribution of outer membrane proteins to the core exoproteome in terms of protein amounts was relatively high. A similar trend was also observed for the variable exoproteome, although in this case the amounts of periplasmic proteins were relatively high compared to the respective protein identifications (Fig. 4D). Nonetheless, the ECPs were the most abundant representatives in the variable exoproteome fraction.

**FIG 5:**
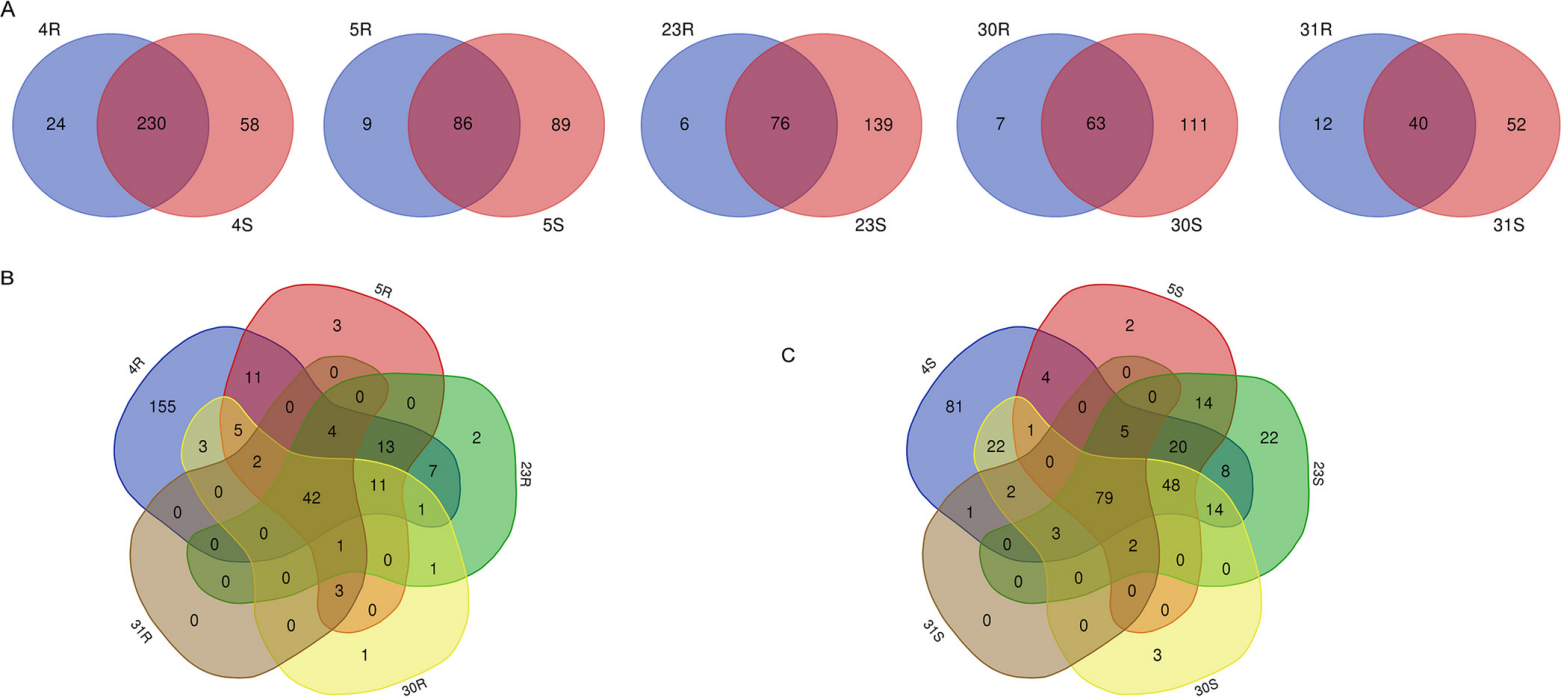
Venn diagrams showing the numbers of identical and uniquely identified extracellular proteins for the different investigated A. actinomycetemcomitans R isolates and the corresponding S strains. (A) Pairwise comparisons for the R isolates and their respective derivative S strains. (B) Comparisons for all R isolates. (C) Comparisons for all S strains collected in the present study.

To visualize the differences in extracellular proteins of R isolates and the respective derivative S strains, Venn diagrams were used. As indicated above, the total numbers of identified extracellular proteins of S strains are higher than those of R isolates, and accordingly, we identified more unique proteins for S strains than for the R isolates. This is particularly evident for the 23S and 30S strains (Fig. 5A). Furthermore, it is noteworthy that a comparison of the identified extracellular proteins of all R isolates revealed 42 common proteins, while these isolates showed variable numbers of unique extracellular proteins (Fig. 5B). In most cases, only few or no unique proteins were identified for the R isolates, the exception being the 4R isolate with 155 unique proteins (Fig. 5B). Among the S strains, we identified 79 common extracellular proteins, and also here, uniquely identified proteins were relatively rare with the exception of the 4S strain, for which 81 unique extracellular proteins were identified (Fig. 5C).

The A. actinomycetemcomitans type strain ATCC 29522 shows the S phenotype. To visualize similarities and differences with the here-investigated clinical R isolates and derivative S strains, Venn diagrams were created including the ATCC 29522 strain. As shown in Fig. S2, ATCC 29522 and the presently investigated R isolates and S strains had a high number of extracellular proteins in common, which was especially evident for 4R and 4S. In contrast, 31R and 31S shared only 37 extracellular proteins with the ATCC 29522 strain (Fig. S2A). Multiple comparisons between all S strains revealed that the 31S strain shared the lowest number of commonly identified proteins with the other S strains, including the ATCC 29522 strain (Fig. S2B).

10.1128/msystems.00254-22.2 FIG S2 Venn diagrams showing the numbers of the identified extracellular proteins of A. actinomycetemcomitans. (A) Comparisons of the numbers of identified extracellular proteins of R isolates of A. actinomycetemcomitans, their respective derivative S strains, and the ATCC 29522 type strain. (B) Multiple comparisons of the numbers of extracellular proteins identified for the different investigated S strains. Download FIG S2, PDF file, 1.5 MB. Copyright © 2022 Fu et al. 2022 Fu et al. https://creativecommons.org/licenses/by/4.0/ This content is distributed under the terms of the Creative Commons Attribution 4.0 International license.

**FIG 6:**
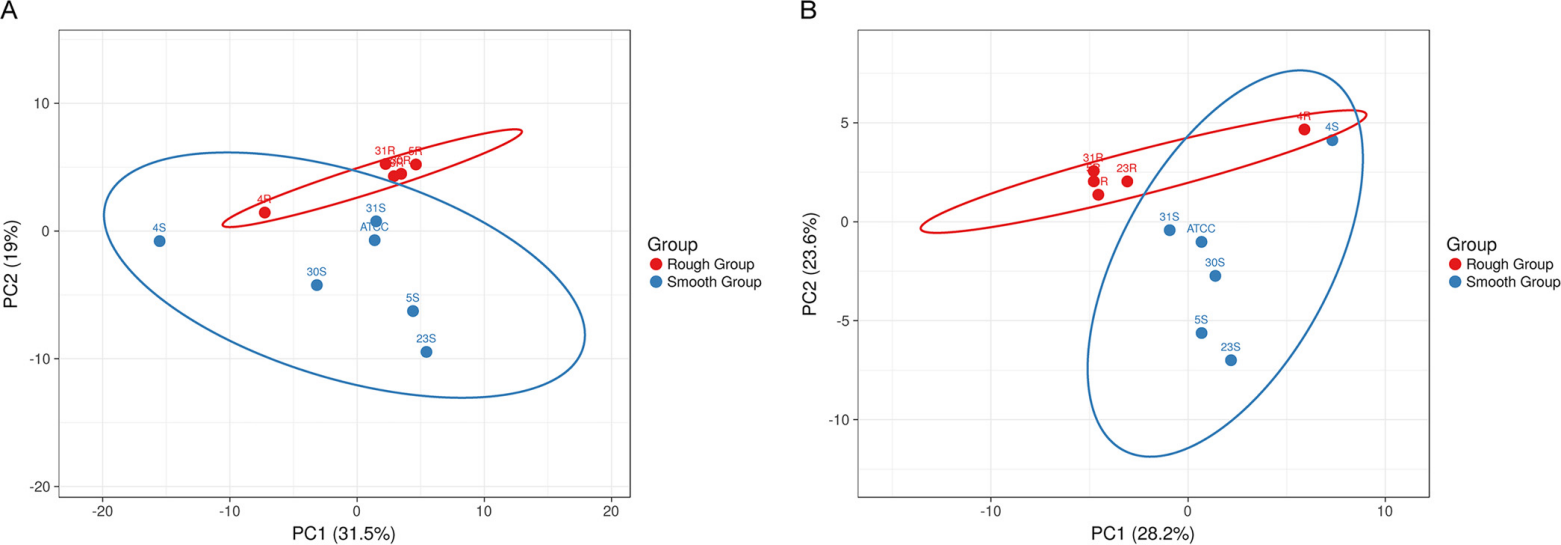
Principal-component analysis (PCA) of identified extracellular proteins of the investigated A. actinomycetemcomitans R isolates and S strains. The PCA plots are based on the NSAF values of extracellular proteins identified for the R and S groups. (A) All identified extracellular proteins; (B) all identified extracellular proteins except the extracellular cytoplasmic proteins (ECPs).

A principal-component analysis (PCA) was performed to compare the exoproteome relationships among the R isolates and the derivative S strains. As shown in Fig. 6, this revealed that the exoproteomes of the investigated S strains are more heterogeneous than those of the R isolates, where the 4R isolate is most distantly positioned in the PCA plot. Likewise, the exoproteome of the 4S strain is more distinct from the exoproteomes of the other investigated S strains. Judged by the PCA analysis, the exoproteome of the 31S strain is most similar to that of the ATCC 29522 type strain and the 30S strain. Subsequently, we also assessed to what extent the exoproteome relationships are influenced by ECPs, considering the huge number of identified ECPs. Interestingly, even without the ECPs, the S strains still showed a higher exoproteome heterogeneity than the R isolates (Fig. 6B). Also in this case, the exoproteome of the 4R isolate was apparently rather distinct from that of the other R isolates. The same was true for the exoproteome of the 4S strain, which differed substantially from the exoproteomes of the other S strains. Based on these observations, we conclude that all extracellular proteins contribute to the distinctive exoproteome profiles of the R isolates and S strains.

**FIG 7:**
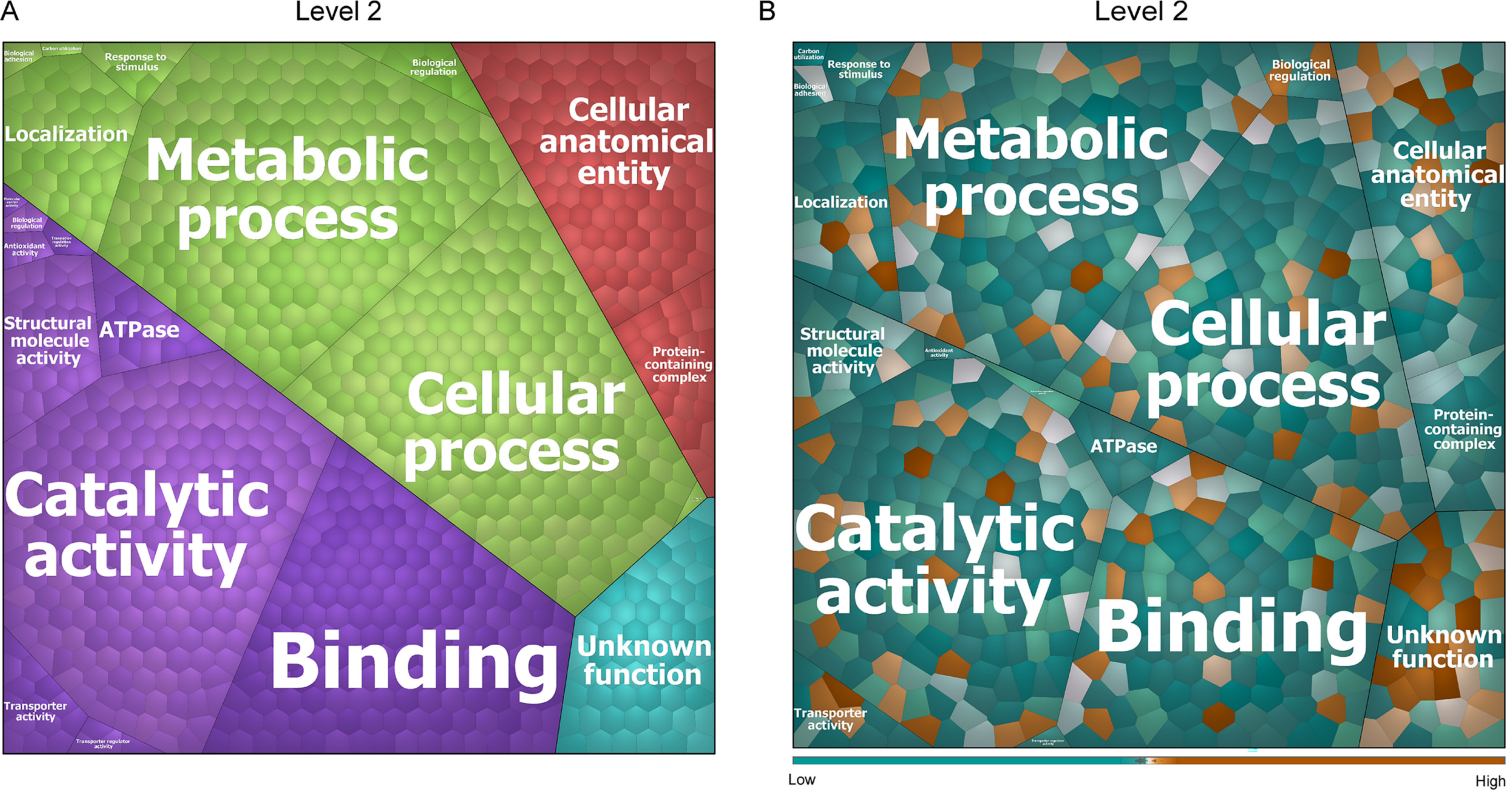
Voronoi treemaps representing the functions of extracellular proteins of all investigated A. actinomycetemcomitans R isolates and S strains. The functions of identified extracellular proteins were evaluated based on GO terms. The treemaps in panels A and B display the level 2 “sublevel functions” in GO terms. (A) Functional categories of identified proteins are represented in different colors. The size of each functional category is proportional to the number of identified proteins belonging to the respective functional category. (B) Relative abundance of all identified extracellular proteins of all R isolates compared to all derivative S strains. The color code represents the relative abundance of the identified proteins based on NSAF values. Orange indicates extracellular proteins with higher abundance in the R isolates, while blue indicates extracellular proteins with higher abundance in the S strains.

To address the functions of the identified extracellular proteins of A. actinomycetemcomitans, the respective gene ontology (GO) terms were obtained using InterPro. This allowed us to group the 351 identified proteins into four top-level functions (Fig. S3A) and 20 second-level functions (Fig. 7A). As shown in the Voronoi treemap of Fig. 7A, the majority of all identified extracellular proteins are involved in substrate binding, catalytic activity, metabolism, cellular processes, and cellular anatomy. The most abundant extracellular proteins of the R and S groups are the porin OmpA, the RTX family leukotoxin LtxA, the TonB-dependent receptor (TonB), the outer membrane lipoprotein Pcp, and the elongation factor Tu (Tuf). These proteins, which belong to the core extracellular proteins that we identified, are involved in porin activity, calcium binding, translational elongation, GTP binding, translation elongation factor activity, and GTPase activity. The relative abundances of all identified extracellular proteins of the R isolates compared to the derivative S strains are schematically represented in the Voronoi treemap of Fig. 7B, with the respective protein names given in Fig. S3B. The most noticeable finding here is that the relative abundances of most of the identified extracellular proteins seem to be higher in the S strains than in the original R isolates. Furthermore, the most abundant extracellular proteins of the R isolates relate to catalytic activity, while the most abundant extracellular proteins of the S strains relate to metabolic processes. Importantly, compared with strains in the S group, the top six most abundant extracellular proteins that we identified among the R isolates are the Flp pilus assembly protein TadF, the outer membrane lipoprotein chaperone LolA, the Flp pilus assembly protein TadD, an OmpW family protein (OmpW), a redoxin family protein (GenBank accession no. PRK14018), and the Flp pilus assembly protein TadG. Of these proteins, only the redoxin family protein (PRK14018) is not a known virulence factor. Although the functions of TadF and TadG are “unknown” according to GO terms, it has been shown that these proteins of A. actinomycetemcomitans have a role in pilus assembly (42, 43). Some other important and well-known virulence factors of A. actinomycetemcomitans are also more abundantly identified among the isolates in the R group. These include the autotransporter adhesin Aae, the cytolethal distending toxin subunit Aa-CdtA (CdtA), the toxin PezT, the cytolethal distending toxin nuclease subunit Aa-CdtB, and OmpA. Compared to the R isolates, the aspartate-ammonia ligase (AsnA), the periplasmic spermidine/putrescine-binding protein 2 (PotD-A), the superoxide dismutase family protein SodC, the 30S ribosomal protein S3 (RpsC), and the 30S ribosomal protein S1 (RpoA) are the top five most abundant extracellular proteins among the S strains. Only SodC has been described as a virulence factor (44).

10.1128/msystems.00254-22.3 FIG S3 Voronoi treemaps for extracellular proteins of all investigated A. actinomycetemcomitans R isolates and S strains. The functions of identified extracellular proteins were evaluated based on GO terms. (A) Top-level functional categories of all identified proteins; (B) the respective protein names. Download FIG S3, PDF file, 0.4 MB. Copyright © 2022 Fu et al. 2022 Fu et al. https://creativecommons.org/licenses/by/4.0/ This content is distributed under the terms of the Creative Commons Attribution 4.0 International license.

### Distinctive virulence factor expression in rough and smooth strains.

**FIG 8:**
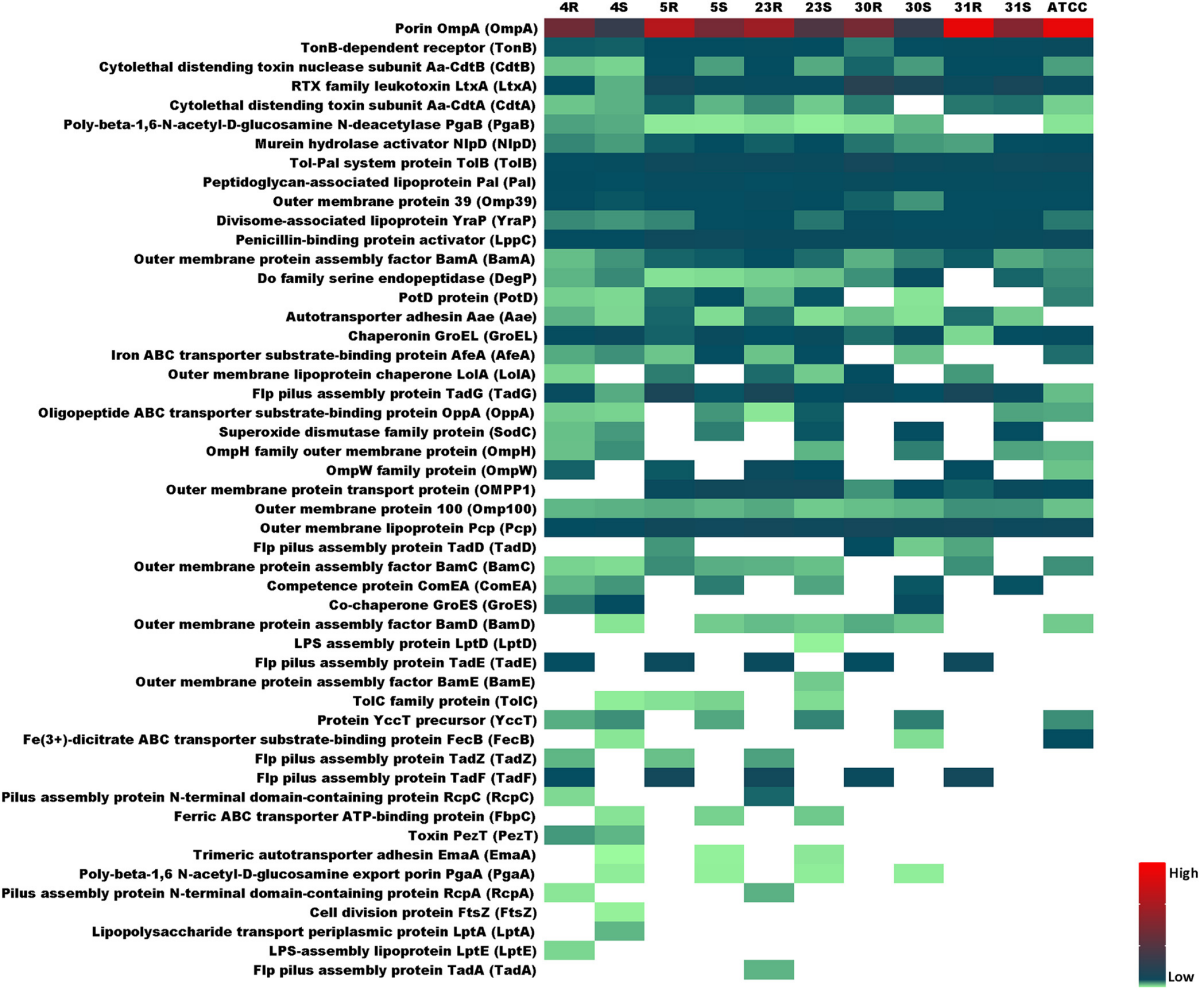
Heat map showing the abundance of known extracellular virulence factors of the investigated A. actinomycetemcomitans R isolates and the respective S strains. A total number of 50 known A. actinomycetemcomitans virulence factors was identified for the investigated A. actinomycetemcomitans R isolates and corresponding S strains. The relative abundance of the identified virulence factors was determined based on normalized spectral counts and is indicated by color-coded bars.

To visualize the relative abundance and isolate/strain-specific differences in the identified A. actinomycetemcomitans virulence factors, the heat map shown in Fig. 8 was created. A total of 50 extracellular virulence factors was identified for the investigated R isolates and the respective S strains. These virulence factors are involved in bacterial adhesion, cytotoxicity, biofilm formation, and immune evasion (45). Interestingly, several extracellular proteins that are assembled into protein complexes were identified in this study, including the CDT, Tad, PGA, β-barrel assembly machinery (BAM) and lipopolysaccharide transport (LPT) proteins. Fifteen of the identified extracellular virulence factors are expressed in all investigated strains, including the well-known virulence factors OmpA (also referred to as Omp29 or Omp34), LtxA, CdtB, Pcp, and Pal. OmpA is actually the most abundantly identified extracellular protein in all 11 investigated A. actinomycetemcomitans isolates and derivative strains, consistent with a previous study (46). Although some other proteins are expressed in all investigated A. actinomycetemcomitans strains, their extracellular abundance varies greatly. For instance, although GroEL and TadG can be identified in the medium of all investigated A. actinomycetemcomitans strains, GroEL was more abundant in the growth medium of the S strains while TadG was more abundant in the growth medium of the R isolates. Other proteins were specifically identified in particular R isolates or S strains. This is exemplified by Aae, which was identified only for clinical R isolates and the derivative S strains, not in the ATCC 29522 type strain. Furthermore, Aae was identified with significantly higher abundance in the medium of the 5R, 23R, and 31R isolates than in the medium of the respective S strains. Of note, some virulence factors were identified exclusively in medium of the R isolates (e.g., TadE, TadF, and TadZ), while other proteins were identified exclusively in the medium of S strains (e.g., EmaA and PagA). An intriguing observation is that the growth media of the 4R, 4S, and 23S strains harbor 37 known virulence factors, while the growth media of other strains contain fewer virulence factors, as exemplified for the 31S strain, for which 24 known virulence factors were identified. For example, SodC and ComEA can be identified in the medium of all derivative S strains, but these proteins were also identified in the medium of the 4R isolate. Furthermore, LolA was identified in the medium of all R isolates, but it was also identified in the medium of the 23S strain. Some proteins were identified only in the media of one or two A. actinomycetemcomitans strains, which is mostly the case for the 4R isolate and the 4S and 23S strains. For instance, LptD and BamE were identified only in the exoproteome of the 23S strain, while LptE was identified only in the exoproteome of the 4R isolate, and FtsZ and LptA were identified only in the exoproteome of the 4S strain. PezT was identified only in media of the 4R isolate and the respective 4S strain. Lastly, although the 23R isolate was found to produce 33 extracellular virulence factors, the virulence factor TadA was uniquely identified in the exoproteome of this isolate.

As exemplified by different Tad proteins, some virulence factors were not identified in the exoproteomes of particular strains. Since this might relate to the loss of the respective genes during the conversion from the R to the S phenotype, we verified whether these genes were still present. To this end, we compared the genome sequences of the here-investigated R isolates and S strains. The results showed that for most proteins detected in the exoproteomes of R isolates but not in those of S strains, the respective genes were present in the S strains. This suggests that these genes were not expressed under the present experimental conditions or were expressed at only very low levels. The exceptions were the 10 aforementioned genes, from *flp-1* to *tadC*, that were absent from the 31S strain and which were apparently lost during the conversion from the R to the S phenotype. The *pezT* gene and, consequently, the respective PezT protein were not detected in the 30R and 31R isolates and the respective 30S and 31S strains.

### A. actinomycetemcomitans pathogenesis in Galleria mellonella.

**FIG 9:**
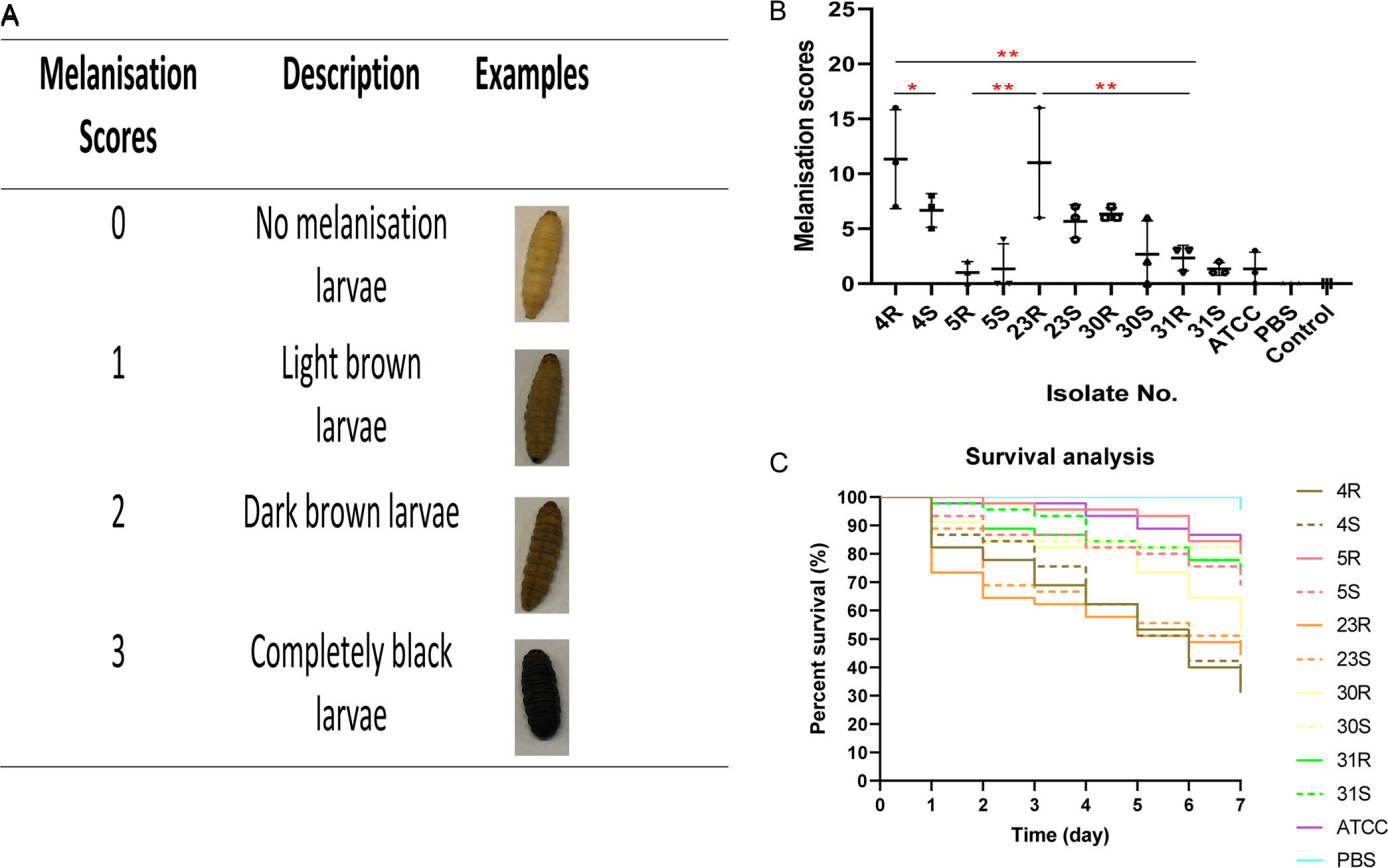
Pathogenicity of A. actinomycetemcomitans R isolates and S strains in a Galleria mellonella larval infection model. Upon injection of an A. actinomycetemcomitans bacterial inoculum of 10 μL, the larvae were incubated at 37°C in the dark and their melanization and viability were monitored over time. Negative control groups consisted of larvae injected with 10 μL PBS (PBS) or untreated larvae (control). (A) Representative images of different degrees of larval melanization and the respective attributed scores. (B) Larval melanization on day 1 postinjection with different A. actinomycetemcomitans R isolates and the corresponding S strains or ATCC 29522 (ATCC). Statistically significant differences were assessed with the Mann-Whitney U test between two unpaired groups and Kruskal-Wallis tests with subsequent Dunn’s or Dunnett’s multiple-comparison tests (*, *P* < 0.03; **, *P* < 0.002; ***, <0.0002; ****, <0.0001). (C) Survival rates of larvae injected with A. actinomycetemcomitans (R isolates and S strains are marked in color code). The *P* values for differences in melanization (B) and survival rates (C) are presented in Table S3.

Since substantial differences in the exoproteomes and extracellular virulence factors were observed between the R isolates and S strains, we wondered to what extent these differences could influence their virulence. To answer this question, a Galleria mellonella larval infection model was used. Since melanization is a key step in the antimicrobial response of G. mellonella larvae upon pathogen injection (47), we first assessed the virulence of different A. actinomycetemcomitans isolates and derivative strains based on melanization and, subsequently, the virulence based on survival rates after injection. Each A. actinomycetemcomitans isolate and strain was injected into 15 larvae (10^7^ CFU/larvae), and three biological replicate analyses were performed. For controls, 15 larvae were injected with sterile phosphate-buffered saline (PBS), and 15 larvae remained untreated. Since infected larvae started to die at 24 h postinjection, melanization was monitored only at this time point. In the PBS control group and the untreated control group, no melanization was observed at 24 h. Larvae infected with the 5R and 31R isolates or the respective 5S and 31S strains always showed light melanization. For comparisons of the virulence assessment based on melanization and determination of the statistical significance of the observed differences, we divided all isolates and strains into three groups, namely, R isolates and their derivative S strains, which we refer to as the R and S groups. This showed that the 4R isolate triggered significantly higher melanization than the 4S strain and the 31R isolate. The 23R isolate showed significantly higher melanization than the 5R and 31R isolates (Fig. 9B).

10.1128/msystems.00254-22.6 TABLE S3 Statistical analysis of differences in the virulence of A. actinomycetemcomitans in the G. mellonella and cell infection models. Download Table S3, XLSX file, 0.02 MB. Copyright © 2022 Fu et al. 2022 Fu et al. https://creativecommons.org/licenses/by/4.0/ This content is distributed under the terms of the Creative Commons Attribution 4.0 International license.

In addition to melanization, the survival of G. mellonella larvae was monitored every 24 h over a period of 7 days. All larvae in the two control groups survived during this period of time. Further, no difference was observed upon larval infection with R isolates and their derivative S strains. However, the 4R isolate appeared to be more pathogenic to G. mellonella larvae than the 5R, 30R, and 31R isolates. Also, the 23R isolate appeared to be more pathogenic to G. mellonella larvae than the 5R and 31R isolates. The 30R isolate was significantly more pathogenic to G. mellonella larvae than the 5R isolate. In the S group, the 4S and 23S strains showed a higher virulence than other S strains. Based on these observations, we conclude that the virulence of A. actinomycetemcomitans does not drastically change upon the transition from the R phenotype to the S phenotype and that the 4R and 23R isolates as well as their derivative S strains always showed higher pathogenesis, at least in the G. mellonella infection model.

### Cytotoxicity of A. actinomycetemcomitans in different human cell infection models.

**FIG 10:**
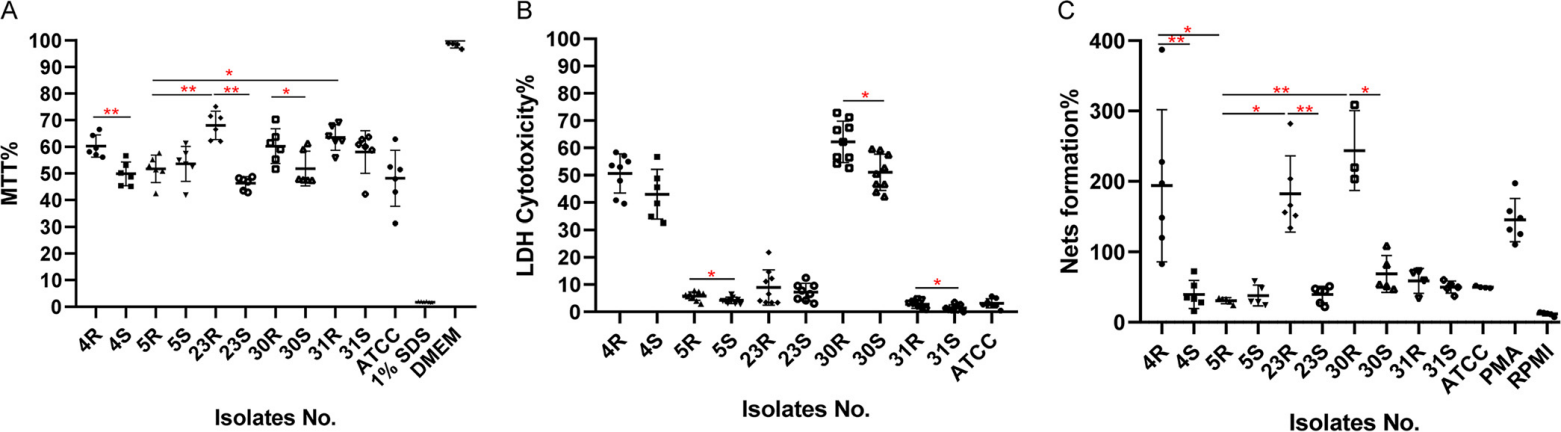
Cytotoxicity of A. actinomycetemcomitans in different human cell infection models. (A) The viability of A253 epithelial cells infected with A. actinomycetemcomitans R isolates or S strains (MOI = 100) was assessed by determining MTT reduction at 6 h postinfection. For a control, the cells were either killed with 1% SDS or supplemented with fresh DMEM. (B and C) Human neutrophils were infected with A. actinomycetemcomitans (MOI = 100), and at 2 h postinfection, neutrophil lysis was evaluated by measuring the release of LDH (B) and NET formation was evaluated by measuring DNA release by high-throughput microscopy (C). For the assessment of NETosis in panel C, NETosis was induced with 0.125 μg/mL (20 nM) PMA. Fresh RPMI medium was added as a negative control. Statistically significant differences were assessed with Kruskal-Wallis tests with subsequent Dunn’s or Dunnett’s multiple-comparison tests (*, *P* < 0.03; **, *P* < 0.002).

To further evaluate the virulence of the R isolates and their derivative S strains, we employed infection models based on human salivary gland epithelial cells and neutrophils. In the epithelial cell infection model, the cells were infected for 6 h with PBS-diluted A. actinomycetemcomitans. Subsequently, the viability of the epithelial cells was measured by measuring the reduction of 3-(4,5-dimethylthiazol-2-yl)-2,5-diphenyltetrazolium bromide (MTT). As shown in Fig. 10A, the 4S, 23S, and 30S strains showed significantly higher cytotoxicity than their parental R isolates. Furthermore, the 5R isolate was significantly more toxic for the epithelial cells than the 23R and 31R isolates. Notably, no significant differences in viability were observed among the S strains. Interestingly, a very different outcome was observed in the neutrophil infection model. In this case, the neutrophils were infected with PBS-diluted A. actinomycetemcomitans for 2 h, and cytotoxicity was measured by determining the amounts of released lactate dehydrogenase (LDH). As shown in Fig. 10B, the 5R, 30R, and 31R isolates exerted significantly higher cytotoxicity toward neutrophils than their derivative S strains. The 4R and 30R isolates were also more toxic than other R isolates. Further, the 4S strain showed a higher cytotoxicity than the 31S and ATCC 29522 strains, while the 30S strain showed higher cytotoxicity than the 5S, 31S, and ATCC 29522 strains.

Importantly, neutrophils may respond to bacterial presence by the formation of so-called neutrophil extracellular traps (NETs) to capture and kill the bacteria. Therefore, we also investigated the formation of NETs upon neutrophil exposure to the different A. actinomycetemcomitans R isolates and S strains by employing a high-throughput NETosis assay. Interestingly, the NET formation followed a pattern that was distinct from the pattern of neutrophil lysis as measured by LDH release. As shown in Fig. 10C, the 4R, 23R, and 30R isolates induced an extensive formation of NETs that trended even higher than observed upon induction of NETosis with 0.125 μg/mL (20 nM) phorbol 12-myristate 13-acetate (PMA). In contrast, the investigated S strains triggered NETosis to a very minor extent, as was the case for the 5R and 31R isolates. Taken together, these observations show that different clinical R isolates of A. actinomycetemcomitans and their derivative S strains display differential cytotoxicity toward human epithelial cells and neutrophils and also elicit differential responses in innate immune cells with respect to the formation of NETs.

### Correlation of A. actinomycetemcomitans virulence and different models with extracellular virulence factors.

To investigate whether the abundance of particular virulence factors is correlated with the virulence of A. actinomycetemcomitans as measured in the five different infection assays (i.e., G. mellonella melanization or killing, killing of epithelial cells, neutrophil lysis, and NETosis), we calculated the statistically significant differences in abundance of virulence factors in the A. actinomycetemcomitans bacteria with high or low virulence in each assay. The respective data are summarized in Table 1 and detailed in Table S4. In addition, we assessed whether the presence or absence of particular virulence factors could be associated with virulence in the five infection assays. In the G. mellonella larval infection model, the presence of the pilus assembly protein N-terminal domain-containing protein RcpA and the pilus assembly protein N-terminal domain-containing protein RcpC was consistently higher in A. actinomycetemcomitans bacteria that induced higher melanization. Furthermore, the poly-beta-1,6-*N-acetyl-*d-glucosamine *N*-deacetylase PgaB and the oligopeptide ABC transporter substrate-binding protein OppA were detectable or produced to higher levels in the A. actinomycetemcomitans bacteria that showed higher G. mellonella killing rates. In the epithelial cell infection model, the chaperonin GroEL was detected at higher levels in the more virulent A. actinomycetemcomitans bacteria, and the TolC family protein TolC was detected in the more virulent A. actinomycetemcomitans bacteria. In neutrophil killing and NETosis, the Do family serine endopeptidase (DegP) was more abundant in the A. actinomycetemcomitans bacteria with higher virulence. Furthermore, the TadE and TadF proteins were detected in the R isolates of A. actinomycetemcomitans with higher virulence toward neutrophils, and the TadG protein was more abundant in these R isolates.

**TABLE 1:** Extracellular virulence factors of A. actinomycetemcomitans significantly or uniquely associated with virulence in different infection models

Infection model	Protein name
Larval melanization	Pilus assembly protein N-terminal domain-containing protein RcpA
Larval survival	Pilus assembly protein N-terminal domain-containing protein RcpC
	Poly-beta-1,6-*N*-acetyl-d-glucosamine *N*-deacetylase PgaB
	Oligopeptide ABC transporter substrate-binding protein OppA
Epithelial cell killing	Chaperonin GroEL
	TolC family protein
Neutrophil killing/NETosis	Do family serine endopeptidase DegP
	Flp pilus assembly protein TadE
	Flp pilus assembly protein TadF
	Flp pilus assembly protein TadG

10.1128/msystems.00254-22.7 TABLE S4 Extracellular virulence factors of A. actinomycetemcomitans bacteria significantly or uniquely associated with virulence in the different infection models. Symbols: ↑, higher abundance of a particular protein; ↓, lower abundance of a particular protein; +, presence of a protein; −, absence of a protein. Download Table S4, XLSX file, 0.02 MB. Copyright © 2022 Fu et al. 2022 Fu et al. https://creativecommons.org/licenses/by/4.0/ This content is distributed under the terms of the Creative Commons Attribution 4.0 International license.

## DISCUSSION

The present study was aimed at comparing the exoproteomes of different serotype b clinical isolates of A. actinomycetemcomitans with the R phenotype and the respective derivative strains with the S phenotype and at analyzing the implications of the observed differences for the infectious behavior of these strains in different model systems.

Phase variations and shifts in colony morphology with implications for virulence have been reported for many bacterial pathogens, the classical example being the rough and smooth phenotypes of the human pathogen Streptococcus pneumoniae (48). The R-to-S conversion in A. actinomycetemcomitans is a spontaneous process that was previously associated with mutations in the *flp* promoter region for the expression of fimbriae (41). Accordingly, the reverse conversion from the S to the R phenotype has so far not been reported. Only one study reported that S strains can reexpress fimbriae when grown on a low-humidity solid medium, but the colonies did not have the star-shaped appearance, which is an important characteristic of the R isolates of A. actinomycetemcomitans (49). In our present study, we also detected mutations at different positions in the −10 region of the *flp* promoter, some of which are the same as previously reported (41). Furthermore, another mutation was found in the *recJ* gene of the 31S strain. Interestingly, in a previous study on Neisseria gonorrhoeae, it has been reported that mutations in *recJ* influenced the expression of pili (50). However, a more likely explanation for the S phenotype of the 31S strain is that this strain had lost most of the *flp*-*tad* locus, which by definition leads to a defimbriated phenotype. As verified by TEM, both the identified *flp* promoter mutations and the deletion in the *flp*-*tad* locus severely affected the fimbriation of A. actinomycetemcomitans, explaining the observed phenotypic R-to-S conversion. Intriguingly, the loss of fimbriation was associated with major changes in protein export by A. actinomycetemcomitans, as initially observed for LtxA by Western blotting and subsequently by our exoproteome analyses. These novel observations were the prime incentive to further investigate the impact of the R-to-S conversions on the virulence of A. actinomycetemcomitans.

A few previous publications addressed the differences between clinical R isolates and their derivative S strains, especially with respect to virulence (12, 41). In contrast, even though S strains of A. actinomycetemcomitans have not been isolated directly from patients, most studies have so far focused on S strains. This relates to the fact that these strains grow homogeneously and do not aggregate or adhere to the culture vessels, which complicates the analysis of the R isolates (7, 51). Furthermore, previous studies addressed the virulence of S strains exclusively *in vitro* (7). Consequently, it was thus far not known to what extent different R isolates and their respective S strains differ in terms of overall production of virulence factors and how these differences impact the overall virulence toward immune cells, epithelial cells, and in *in vivo* small animal models. Therefore, in the present study, we compared the exoproteome profiles and infectious behavior of five clinical serotype b A. actinomycetemcomitans isolates with the R phenotype and their derivative S strains, as well as the reference strain ATCC 29522, which also has the S phenotype.

The serotype b strains investigated in the present study all have the non-JP2 genotype, and they are all *cagE* positive. Previous research associated the presence of the *cagE* gene with high leukotoxicity in A. actinomycetemcomitans, including the JP2 and non-JP2 genotypes (32). Nonetheless, in our present study, we observed strong variations in the levels of LtxA secreted by the different *cagE*-positive isolates. This is in accordance with previous observations by Nørskov-Lauritsen et al. (52), who showed that LtxA production varies substantially in all genotypes of A. actinomycetemcomitans. On the other hand, our findings imply that LtxA secretion does not strictly correlate with the presence of *cagE*. However, it is presently not clear whether our investigated A. actinomycetemcomitans strains expressed *cagE*, since the CagE protein remained undetected in our proteome analyses. Furthermore, it was previously proposed that S strains could secrete more LtxA than R isolates, which might be related to changes in the pH of the growth medium and the age of the culture (15). For instance, culture medium with an initial pH of 7.1 was reported to result in the secretion of higher amounts of LtxA than culture medium with a higher initial pH, while young cultures of A. actinomycetemcomitans were reported to yield higher levels of secreted LtxA than old cultures (15). In another study, it was observed that LtxA is released from the A. actinomycetemcomitans bacteria in the presence of serum (53). Therefore, we used BHI broth for our present study with a pH of 7.4, where most LtxA is released from the bacteria, and we ensured that all strains were grown in parallel. Nonetheless, we observed by mass spectrometry that the investigated R isolates generally secreted more LtxA than the respective S strains. At present, we do not know the reason for these different observations, but our findings imply that the *cagE* genotype, growth medium pH, and age of the culture are not the only determinants for LtxA secretion. Instead, the R or S phenotype seems more important, at least as measured by mass spectrometry under the present culture conditions. Here, it should be noted that it is not directly evident from our Western blotting experiments that the R isolates secreted more LtxA than the S isolates, except for the 31R-31S pair. However, while Western blotting visualizes mostly the intact LtxA and some dominant degradation products, this distinction is not made in the mass spectrometric analyses, since mass spectrometry detects all LtxA-specific peptides and does not discriminate between intact and partially degraded forms of LtxA. Possibly, this difference in LtxA detection also relates to the use of an epitope-specific monoclonal antibody against LtxA for the Western blotting experiments.

Overall, we observed significant differences between the exoproteomes of the investigated R isolates and S strains of A. actinomycetemcomitans, with the S strains secreting significantly more proteins than the R isolates. This is mostly related to the higher numbers of extracellular cytoplasmic proteins, the so-called ECPs, that were detected for the S strains. The release of ECPs into the growth medium is not exclusive for A. actinomycetemcomitans, as this phenomenon has also been observed for many other microorganisms (37, 54, 55). For instance, in our previous exoproteome profiling studies with clinical isolates of S. aureus or Porphyromonas gingivalis, we also observed many ECPs. Interestingly, this phenomenon is not limited to clinical isolates of pathogens but is also observed for bacteria like Bacillus subtilis that are used for the industrial production of secreted proteins (56). This release of ECPs into the extracellular milieu has been attributed to the activity of prophages, autolysins, and toxins that weaken the bacterial membrane (56–58). Furthermore, the amounts of ECPs were shown to be inversely correlated to protease secretion, where secreted proteases are believed to degrade most of the ECPs (56). In the present study, we show that a reduced fimbriation of A. actinomycetemcomitans, due either to promoter mutations or loss of the respective genes, is associated with elevated levels of ECPs. This implies that the fimbriae somehow prevent the release of ECPs into the growth medium or that their absence somehow weakens the cell envelope of A. actinomycetemcomitans. In this respect, it is noteworthy that the loss of fimbriae also reduced the cellular retention of the virulence factor LtxA, as shown by Western blotting for the 30S and 31S strains. Additionally, our proteome analyses show that the murein hydrolase activator NlpD was present in elevated amounts in the growth medium of four of the six investigated S strains, which suggests that in these strains, the elevated levels of ECPs could also be enhanced by cell wall weakening. In fact, murein hydrolase activity might contribute to the observed transition from the R to the S phenotype, as it could potentially affect the separation of different bacterial cells that cluster together. The loss of cell clustering should, however, be attributed primarily to the reduced fimbriation of the S isolates. The latter view is supported by our TEM analyses and the fact that proteins involved in Flp pilus assembly (e.g., TadE and TadF) were no longer detected in the growth medium of the S strains or were present in reduced amounts (e.g., TadD and TadG). Together, only 34 proteins were detectable in the exoproteomes of all investigated A. actinomycetemcomitans R isolates and S strains, which include the main virulence factors of A. actinomycetemcomitans. Furthermore, these virulence-related proteins were most abundantly detectable in the growth medium of the R isolates, which would be consistent with the observation that only R type A. actinomycetemcomitans is collected from patients with periodontitis.

Importantly, our present study allowed us to evaluate the importance of different virulence factors in different infection models. Insects like G. mellonella possess an innate immune system that resembles the human innate immune system, and accordingly, infection experiments with insect larvae may help us to understand the bacterial pathogenicity (59–61). Our larval infection experiments implicate the RcpA, RcpC, PgaB, and OppA proteins in the virulence of A. actinomycetemcomitans, where RcpA and RcpC are more related to melanization and PgaB is more related to larval killing. RcpA and RcpC, which are encoded by the *tad* locus, are essential for Flp pilus biogenesis, host colonization, autoaggregation, and immunoreactivity (43, 62). PgaB is crucial for exopolysaccharide export and mediates biofilm formation and attachment to cell surfaces, thereby helping A. actinomycetemcomitans to evade host immune defenses (63). A direct role of OppA in virulence has not yet been reported, but the OppA protein of the pathogen Streptococcus suis is an immunogenic surface protein and mutant bacteria lacking *oppA* are affected in growth and attenuated in a murine infection model (64). Our findings suggest that RcpA and RcpB are important for larval colonization by A. actinomycetemcomitans and that PgaB and OppA may help the bacteria evade the innate immune defenses. Our experiments in the epithelial cell infection model indicate a role for GroEL in the virulence of A. actinomycetemcomitans. Indeed, the GroEL protein of A. actinomycetemcomitans was previously shown to stimulate epithelial cell proliferation, and it may be cytotoxic when added in high amounts or upon long-term exposure (65, 66). In addition, GroEL of A. actinomycetemcomitans has been shown to induce apoptosis in primary human T cells (67). TolC is an antimicrobial resistance protein with a role in LtxA secretion by A. actinomycetemcomitans (68). The TolC protein has been shown to have various different functions in other bacteria. In particular, a *tolC* mutant of Francisella tularensis showed higher cytotoxicity and induced a stronger proinflammatory response in macrophages (69). Our present findings indicate that the GroEL and TolC proteins play a role in the cytotoxicity of A. actinomycetemcomitans toward epithelial cells.

LtxA is known to induce the killing of human leukocytes, and especially high concentrations of LtxA help A. actinomycetemcomitans to avoid phagocytosis and killing by neutrophils (70). In our present neutrophil infection models, LtxA may contribute to neutrophil lysis, but its role is not clearly evident and may be overshadowed by other virulence factors. This view is supported by the fact that mutants lacking *ltxA*, or even lacking *ltxA* and all three *cdt* genes, still retained significant cytotoxicity (71). For instance, superoxide dismutase (Sod) may protect both the bacteria and LtxA from reactive oxygen species produced by inflammatory cells of the host (44). However, SodC was detected more abundantly in our S strains, which showed limited virulence toward neutrophils. In contrast, the TadE, TadF, and TadG proteins involved in Flp pilus assembly (11, 72) were associated with neutrophil killing, suggesting that these pili have a role in immune evasion. Intriguingly, while previous studies implicated LtxA in NETosis (73), we did not observe a direct correlation between LtxA and NETosis. In contrast, A. actinomycetemcomitans bacteria producing low levels of LtxA (i.e., 4R) induced more NET formation than strains producing high amounts of LtxA (i.e., 30R). Finally, while DegP was not previously identified in A. actinomycetemcomitans (74), the DegP protein was identified in our present analyses with a potential role in neutrophil killing and NETosis. This finding is consistent with other studies, where, for instance, a *htrA* (*degP*) mutant of Campylobacter jejuni displayed reduced attachment to epithelial cells and caused reduced cellular apoptosis and proinflammatory immune responses in a murine infection model (75).

Considering all this together, we conclude that our present investigations provide a comprehensive overview of the exoproteome of A. actinomycetemcomitans, highlighting significant differences between clinical isolates with the R colony phenotype and their derivative S strains. Importantly, the comparisons of the exoproteome compositions of different R isolates and S strains focus attention on potentially critical virulence factors. However, while the presently identified associations between particular virulence factors of A. actinomycetemcomitans and the behavior of the investigated clinical isolates in our different infection models are highly compelling, it will also be of interest to verify in future studies the individual contributions of these virulence factors to the observed virulence phenotypes with the help of engineered mutant strains that lack the respective genes. Likewise, it would be interesting to assess the levels of the identified virulence factors during infection experiments with larvae of G. mellonella, epithelial cells, or human neutrophils. Such studies will provide further clues to investigate the virulence of A. actinomycetemcomitans and potential preventive or therapeutic avenues to neutralize this important oral pathogen.

## MATERIALS AND METHODS

### Bacterial isolates, growth conditions, and counting of CFU.

The A. actinomycetemcomitans reference strain ATCC 29522 (S type) and 27 clinical R type isolates from Dutch patients with periodontitis were used in the present study. The bacteria were cultured at 5% CO_2_ and 37°C using brain heart infusion (BHI) agar plates or BHI broth (Oxoid) supplemented with 5% l-cysteine, 5 mg/L hemin, and 1 mg/L menadione. BHI broth was inoculated with a 3-day-old colony from BHI agar, and cultures were incubated for 40 h, at which time the cultures of clinical R isolates and the derivative S strains had reached the stationary growth phase with comparable numbers of CFU. For exoproteome analyses, proteins were collected from the growth medium. To avoid contamination of cultures with R type bacteria by spontaneously arising S type bacteria, culturing was always started from frozen stock.

To count CFU, 4-mL culture samples of R type isolates were dispersed with a sonicator (Misonix) using 45-s pulses with intervals of 5 s. The optical density at 600 nm (OD_600_) of sonicated culture samples was measured, and CFU counts were determined by plating, allowing the correlation of OD_600_ with CFU. Likewise, the correlation between OD_600_ and CFU was determined for the S strains, but the sonication step was omitted.

### Species identification and PCR analysis.

A. actinomycetemcomitans isolates were identified by matrix-assisted laser desorption ionization–time of flight (MALDI-TOF) mass spectrometry using a MALDI Biotyper (Bruker Corporation) as previously described (54).

Genomic template DNA of the A. actinomycetemcomitans reference strain and clinical isolates was obtained by the boiling lysis method. The serotypes of A. actinomycetemcomitans isolates were confirmed by PCRs using specific primers as previously described (76). Briefly, the serotypes b, c, and f were identified by multiplex PCR, while serotypes a, d, and e were identified by individual PCR. The amplification conditions involved initial denaturation at 94°C for 5 min, 30 cycles at 94°C for 30 s, 55°C for 30 s, and 72°C for 30 s, and a final extension at 72°C for 5 min. The ATCC 29522 type strain (serotype b) and the sequenced A. actinomycetemcomitans 14R (serotype a), 29R (serotype b), and 1R (serotype c) isolates were used as controls. The identification of serotypes d, e, and f by PCR was verified by sequencing purified PCR products. *itx* genotyping was performed by individual PCR as previously described (76, 77), involving an initial denaturation at 97°C for 4 min, 25 cycles at 95°C for 1 min, 55°C for 1 min, and 72°C for 1 min, and a final extension at 72°C for 10 min. The ATCC 29522 type strain and the A. actinomycetemcomitans 29R isolate were used as controls. Primers used for PCR are shown in Table S5 in the supplemental material.

10.1128/msystems.00254-22.8 TABLE S5 Primers for A. actinomycetemcomitans serotype and genotype assessment by PCR. Download Table S5, XLSX file, 0.01 MB. Copyright © 2022 Fu et al. 2022 Fu et al. https://creativecommons.org/licenses/by/4.0/ This content is distributed under the terms of the Creative Commons Attribution 4.0 International license.

### Transformation of A. actinomycetemcomitans from rough to smooth phenotype.

The investigated A. actinomycetemcomitans R type isolates were transformed to S type strains by passaging in BHI broth. Briefly, a single R type colony was picked from BHI agar plates and cultured in 5 mL BHI broth overnight. Subsequently, 1-mL aliquots of overnight cultures were used to inoculate 5 mL BHI broth until the bacteria no longer adhered to the tube and cultures appeared completely homogeneous. The collected S type strains were no longer aggregating when cultured in broth.

### Preparation of extracellular proteins and sample preparation for mass spectrometry.

To collect extracellular proteins of A. actinomycetemcomitans, the investigated bacteria were cultured in BHI broth in triplicate as described above. Bacteria were separated from the growth medium by centrifugation, and extracellular proteins were precipitated from the growth medium fraction with trichloroacetic acid (TCA; Sigma-Aldrich, St. Louis, MO, USA) at 4°C overnight. The precipitated proteins were collected by centrifugation, washed once with pure ice-cold acetone, and dried at 60°C. The dried proteins were resuspended in 6 M urea, 0.1 M HEPES, and 0.25 M NaCl, and their concentration was measured using the Pierce bicinchoninic acid (BCA) protein assay kit (Thermo Fisher Scientific), according to the manufacturer’s instructions. Protein samples of 37.5 μg were separated by lithium dodecyl sulfate (LDS)-polyacrylamide gel electrophoresis (PAGE) on NuPAGE gels (Invitrogen), providing a 1-cm separation distance, and stained with SimplyBlue SafeStain (Life Technologies). Subsequently, the proteins were prepared for MS/MS analyses as previously described (78). Briefly, gel lanes were cut, resulting in one gel piece per sample. These gel pieces were then cut into smaller blocks and transferred into low-binding tubes, where they were destained and dried in a vacuum centrifuge prior to the addition of a solution of 0.02 μg/μL trypsin (Promega). Trypsin digestion was carried out at 37°C overnight, and subsequently, peptides were eluted in water by ultrasonication. The peptide-containing supernatant was transferred into a fresh tube and desiccated in a vacuum centrifuge, and peptides were resolubilized in 0.1% (vol/vol) acetic acid for MS/MS analysis.

### LDS-PAGE and Western blotting.

For LDS-PAGE analyses, extracellular protein samples were prepared as described above, separated on NuPAGE gels, and stained with SimplyBlue SafeStain. Cellular protein samples were prepared by collecting the different bacteria by centrifugation and bead-beating with glass beads using a Precellys 24 bead beater (Bertin Technologies). For Western blotting analyses to examine the presence of LtxA in cellular and growth medium fractions, instead of SimplyBlue staining, the separated proteins were semidry blotted from NuPAGE gels to a Protran nitrocellulose transfer membrane (Whatman). Subsequent immunodetection of LtxA was performed using the LtxA-specific monoclonal antibody 83 (40) and an IRDye 800CW-labeled secondary goat anti-mouse antibody. Antibody binding was detected using an Amersham Typhoon biomolecular imager (Cytiva).

### Whole-genome sequencing and SNP analysis.

Whole-genome sequencing of the investigated A. actinomycetemcomitans R type isolates and S strains was performed on a Nanopore MinION and Illumina platform. Subsequently, a hybrid assembly of the Nanopore and Illumina sequence data was performed using SPAdes version 3.11 (http://bioinf.spbau.ru/en/content/spades-311-released) (79). SNP analyses were performed based on short-read sequencing data using Breseq 0.35.7 (80). The sequence analyses showed that the investigated clinical A. actinomycetemcomitans isolates and their derivative strains lacked extrachromosomal elements, such as plasmids.

### Transmission electron microscopy.

A. actinomycetemcomitans bacteria were cultured in 4 mL BHI broth as described above. The bacteria were collected by centrifugation at 8,000 × *g* for 10 min at 4°C, washed twice with PBS, fixed in 200 μL of 4% paraformaldehyde (PFA), and resuspended in 150 μL PBS. Five microliters of the cell pellet was added to a copper grid and subsequently stained with 4% uranyl-acetic acid. After blotting of excess liquid with Whatman filter paper and subsequent air drying of the samples, the presence of fimbriae on the bacteria was inspected by TEM with a Talos F200i electron microscope at 80 kV.

### Mass spectrometry and data processing.

LC-MS/MS analyses were performed on an LTQ Orbitrap XL (Thermo Fisher Scientific) using an EASY-nLC II liquid chromatography system. Tryptic peptides were subjected to liquid chromatography separation by loading them on a self-packed analytical column (outside diameter, 360 μm; length, 20 cm) filled with 3-μm-diameter C_18_ particles (Dr. Maisch HPLC GmbH). Peptides were eluted by a binary nonlinear gradient of 5 to 99% acetonitrile in 0.1% acetic acid over 151 min with a flow rate of 300 nL/min and subsequently subjected to electrospray ionization-based MS. For MS analysis, a full scan in the Orbitrap with a resolution of 30,000 was followed by collision-induced dissociation (CID) of the five most abundant precursor ions. MS/MS experiments were acquired in the linear ion trap.

A nonredundant RAST annotation was performed based on the whole-genome sequences of all investigated A. actinomycetemcomitans bacteria. Protein sequences that differed in only 1 amino acid were included in this database. The resulting nonredundant database of A. actinomycetemcomitans was supplemented with protein sequences of Bos taurus downloaded from UniProt on 5 December 2019 (UniProtKB taxonomy ID 9913), as well as common laboratory contaminants, and a reverse entry for every forward entry was added, resulting in a total of 101,396 entries. Sorcerer-SEQUEST 4 (Sage-N Research) was applied for database searching by using fully specific tryptic cleavage (KR/P) with up to two missed cleavages and methionine oxidation (+15.99 Da) as variable modifications. Precursor mass tolerance was set to 10 ppm, and fragment mass tolerance was set to 0.5 Da. Validation of MS/MS-based peptide and protein identification was performed with Scaffold V4.8.7 (Proteome Software). Peptide identifications were accepted if they exhibited at least deltaCn scores (i.e. normalized score differences between selected peptide spectrum matches and highest-scoring peptide spectrum matches for the respective spectra) of greater than 0.1, and XCorr scores of greater than 2.2, 3.3, and 3.75 for doubly, triply and all higher charged peptides, respectively. Protein identifications were accepted if at least 2 unique peptides were identified in two out of three replicates with the above criteria. With these filter parameters, 0.5% false-positive hits were obtained. For protein quantification, NSAF values (81) were exported. Statistical significance required a *P* value of <0.05 in Student's *t* test. Protein abundances were considered to be differential if the log_2_ fold change was greater than |0.8|. The data were exported from Scaffold to Microsoft Excel for further analysis. For annotation of identified proteins as presented in Table S2, the NCBI identifier was used.

### Assessment of A. actinomycetemcomitans virulence with a Galleria mellonella infection model.

G. mellonella larvae at the final instar stage (Frits Kuiper, Groningen, the Netherlands) were kept on wood shavings at room temperature in the dark. To avoid sampling biases, larvae with any signs of melanization or deformity were rejected, and only larvae of 300 to 350 mg that met the criteria were utilized for infection experiments within 2 days of receipt.

A. actinomycetemcomitans bacteria were collected from BHI medium by centrifugation at 8,000 × *g* for 10 min at 4°C and diluted to the desired number of CFU per mL in PBS. A total of 1 × 10^7^ A. actinomycetemcomitans bacteria in 10 μL PBS were used to inoculate larvae via the last left proleg by use of an insulin pen (HumanPen). After injection, the larvae were kept at 37°C in the dark. Their melanization was monitored after 24 h postinfection, and mortality was monitored every 24 h over 7 days postinfection. Larvae that displayed no movement when they were touched were considered dead. Per experiment, each of the investigated A. actinomycetemcomitans isolates or strains was used to infect 15 larvae and three biological replicates of the larval infections were performed (*n* = 45). For controls, one group (*n* = 15) of larvae was injected with 10 μL of sterile PBS, while another group (*n* = 15) of larvae remained untreated.

### Assessment of A. actinomycetemcomitans cytotoxicity in epithelial cell and neutrophil infection models. (i) MTT assay with epithelial cells.

A253 salivary gland epithelial cells were cultured in Dulbecco’s modified Eagle medium (DMEM)-GlutaMAX (Thermo Fisher Scientific) supplemented with 10% fetal calf serum (Sigma-Aldrich, USA) at 37°C and 5% CO_2_. A total of 10^5^ cells were inoculated in 96-well culture plates and incubated for 24 h. Prior to infection, the wells were washed with PBS to remove nonadherent cells. The cells were infected at a multiplicity of infection (MOI) of 100 with PBS-diluted A. actinomycetemcomitans. Noninternalized bacteria were eliminated 6 h postinfection by three washes with PBS and the subsequent addition of DMEM containing 200 μg/mL of gentamicin. Culturing in the presence of gentamicin was continued for 1 h. Subsequently, internalized bacteria were eliminated by replacing the gentamicin-containing medium with DMEM containing 100 μg/mL of azithromycin, and cultivation was continued for 2 h. The viability of the infected epithelial cells was then evaluated by incubation for 3 h with 0.5 mg/mL 3-(4,5-dimethylthiazol-2-yl)-2,5-diphenyltetrazolium bromide (MTT; Sigma-Aldrich). Lastly, the cells were resuspended in 150 μL of acidic isopropanol to solubilize colored formazan crystals resulting from MTT reduction by mitochondrial activity. The formation of formazan was quantified with a Synergy HT microplate spectrophotometer (BioTek Instruments) by measuring the absorbance at 570 nm, using 620 nm as the background wavelength. The percentage of absorbance for each treated sample was normalized to that of each untreated control.

### (ii) LDH cytotoxicity assay with neutrophils.

Fresh blood donations were received from healthy volunteers (female, aged around 30 years) who had been medically examined. Neutrophils were isolated using Lymphoprep buffer (Stemcell) as previously described (82) and incubated in RPMI medium (Thermo Fisher Scientific) supplemented with 2 mM l-glutamine and 10% donor plasma for 30 min in a humidified incubator (37°C, 5% CO_2_).

To assess neutrophil lysis upon infection with A. actinomycetemcomitans, the release of LDH was evaluated using the LDH cytotoxicity assay kit (Thermo Fisher Scientific) in accordance with the manufacturer’s instructions. In brief, aliquots of 3.5 × 10^4^ neutrophils were resuspended in 100 μL medium as described above in a 96-well tissue culture plate. The neutrophils were then challenged with A. actinomycetemcomitans at an MOI of 100 for 2 h. For a control, the maximal LDH activity was measured by the addition of 10 μL of 10× lysis buffer, and incubation was continued for 45 min. As a control for spontaneous LDH activity, 10 μL MilliQ water was added to neutrophils and incubation was continued for 2 h. Next, 50-μL aliquots of medium of the A. actinomycetemcomitans-treated and control samples were transferred to a new 96-well plate in triplicate and 50 μL of reaction mixture was added to each sample. Upon incubation at room temperature for 30 min in the dark, 50 μL of Stop Solution (Thermo Fisher Scientific) was added and the samples were mixed by gentle tapping. The release of LDH from the neutrophils was evaluated by measuring the absorbance at 490 nm and 680 nm. The percentage of cytotoxicity was calculated by subtracting the 680-nm absorbance value (background signal from instrument) from the 490-nm absorbance value and using the following formula: % cytotoxicity = [(A. actinomycetemcomitans-treated LDH activity − spontaneous LDH activity)/(maximum LDH activity − spontaneous LDH activity)] × 100.

### NET formation.

Fresh human neutrophils were used to assess neutrophil extracellular trap (NET) formation by use of a previously described protocol based on the PKH26 kit (Sigma-Aldrich) with minor modifications (83). In brief, 37,500 neutrophils in RPMI medium were stained with a 2 μM concentration of the red fluorescent cell linker provided in the PKH26 kit before they were challenged with A. actinomycetemcomitans for 2 h. Subsequently, the infected neutrophils were stained with a 5 μM concentration of the membrane-impermeable DNA dye Sytox green (Thermo Fisher Scientific), and the samples were fixed with PFA. Finally, NETs were visualized by immunofluorescence confocal microscopy (Zeiss Celldiscoverer 7). A 0.125-μg/mL (20 nM) concentration of PMA in PRMI medium was added to neutrophils as a positive control for NETosis, and fresh RPMI medium was used as a negative control. All experiments were performed as three biological replicates with triplicate measurements per condition.

### Statistical and bioinformatic analyses.

Protein localization predictions were performed using UniProt, PSORTb (version 3.0.3) (84), ProtComp (version 9.0), and Cell-PLoc (version 2.0) (85). To predict signal sequences, transmembrane alpha-helixes and proteins with a lipobox, SignalP (version 5.0) (86), SecretomeP (version 2.0) (87), TMHMM (version 2.0) (88), and LipoP (version 1.0) (88) were used. Virulence genes were predicted with VirulentPred, which predicts bacterial virulence factors based on their amino acid composition, the Cascased bilayer cascade support vector machine module (SVM) (89), and the Prediction of Pathogenic Proteins in Metagenomic Data Sets (MP3) website (90). Furthermore, subcellular localization predictions and virulence gene predictions were evaluated by manual curation based on published literature. To assess the overall relationships between R type isolates of S strains in terms of their exoproteome profiles, a principal-component analysis (PCA) was performed based on MS data using ClustVis (91). For visualization of protein functions, Voronoi treemaps were built based on gene ontology (GO) terms using Paver (version 2.1) (Decodon GmbH, Greifswald, Germany) (92).

Statistical analyses were performed with GraphPad Prism 8.0.1. Statistical significance of differences between two unpaired groups was assessed with the Mann-Whitney U test. Data sets from cytotoxicity studies with mammalian cells relating to three or more unmatched groups were statistically evaluated with Kruskal-Wallis tests and subsequent Dunn’s or Dunnett’s multiple-comparison tests. The statistical significance of differences in the survival of G. mellonella larvae upon infection with A. actinomycetemcomitans was assessed by log rank (Mantel-Cox) tests. A *P* value of <0.05 was considered statistically significant.

### Biological and chemical safety.

A. actinomycetemcomitans is a biosafety level 2 (BSL-2) microbiological agent and was handled in accordance with appropriate safety procedures. All experiments involving live A. actinomycetemcomitans isolates and chemical manipulations of A. actinomycetemcomitans protein extracts were performed under appropriate containment conditions, and protective gloves were worn. All chemicals and reagents used in the study were handled according to the local guidelines for safe usage and protection of the environment.

### Ethics statement.

Clinical A. actinomycetemcomitans isolates were pseudoanonymously cultured for diagnostic purposes from patients in the Netherlands with destructive periodontal disease and in adherence with the Declaration of Helsinki. Since the present study includes no patient data or potentially identifying information, individual written consent or ethical approval was not required for the present bacteriological analyses. Blood donations from healthy human volunteers were collected based on written informed consent with approval of the medical ethics committee of the University Medical Center Groningen (UMCG; approval no. Metc2012-375) and in accordance with the Declaration of Helsinki guidelines.

### Data availability.

The results of whole-genome sequences were submitted to GenBank (NCBI) through BioProject PRJNA767258 under the accession numbers JAIWZS000000000 (isolate 4R), CP085096 (strain 4S), CP085095 (isolate 5R), CP085094 (strain 5S), JAIWZR000000000 (isolate23R), CP085093 (strain 23S), CP085092 (isolate 30R), CP085091 (strain 30S), CP085090 (isolate 31R), and CP085089 (strain 31S) and JAJHPH000000000 (strain ATCC 29522). The MS data analyzed in this article have been deposited to the ProteomeXchange Consortium via the PRIDE partner repository with the data set identifier PXD028940.
