# Supplementary figures and images for: Connections between Exoproteome Heterogeneity and Virulence in the Oral Pathogen Aggregatibacter actinomycetemcomitans
Source: mSystems. 2022 Jun 13;7(3):e00254-22. doi: 10.1128/msystems.00254-22 (PMC9239275; doi:10.1128/msystems.00254-22)

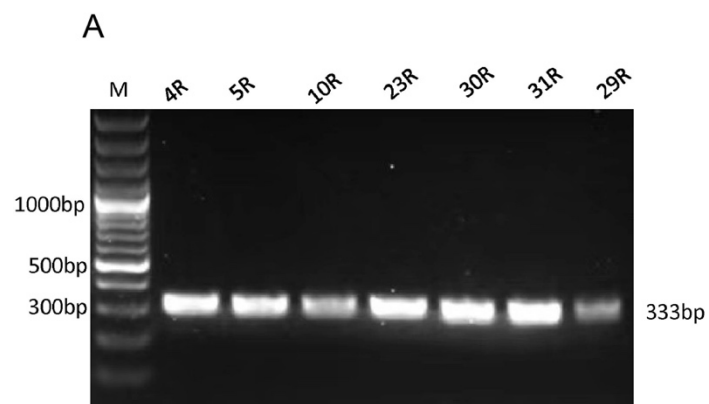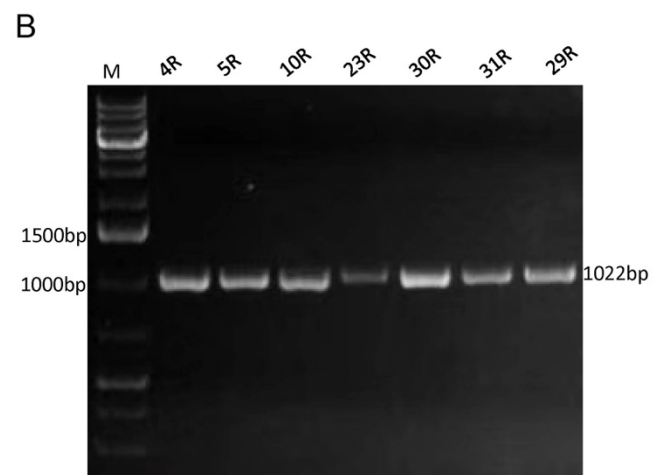

**C**

| Strains | Passages<br>(from rough to smooth) |
|---------|------------------------------------|
| Aa4R    | 14                                 |
| Aa5R    | 12                                 |
| Aa10R   | 12                                 |
| Aa23R   | 6                                  |
| Aa29R   | 10                                 |
| Aa30R   | 11                                 |
| Aa31R   | 8                                  |

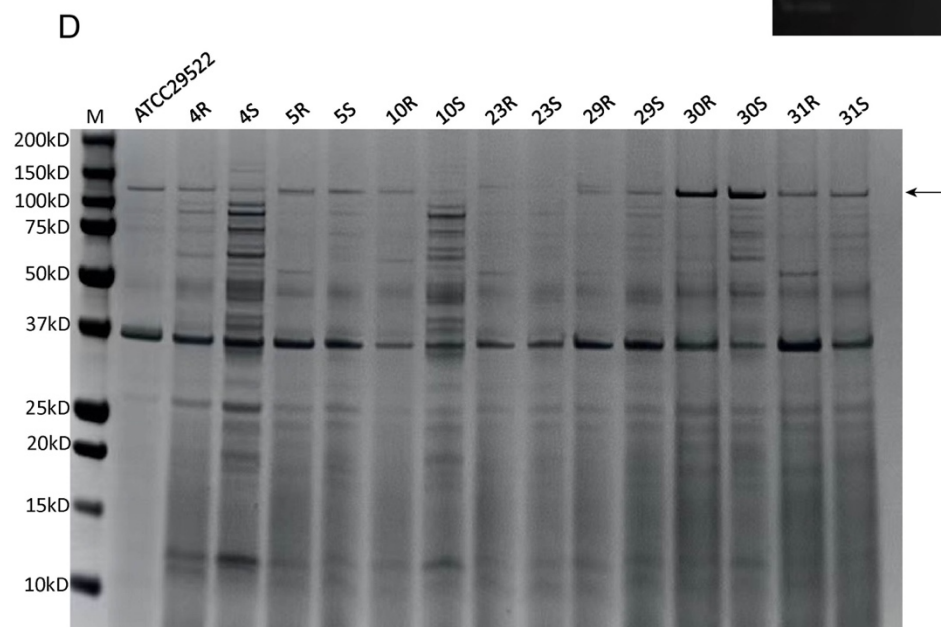

Supplement: FIG S1 [file msystems.00254-22-sf001.pdf]

A

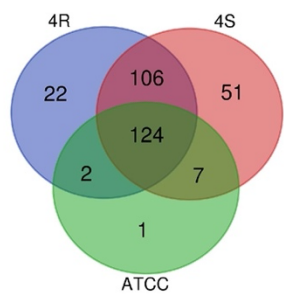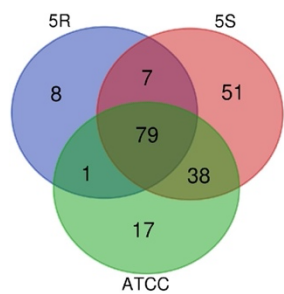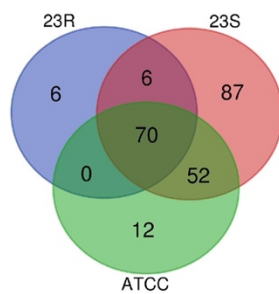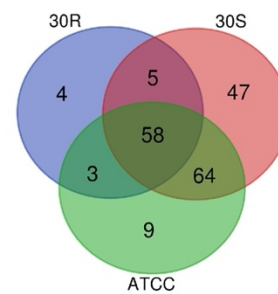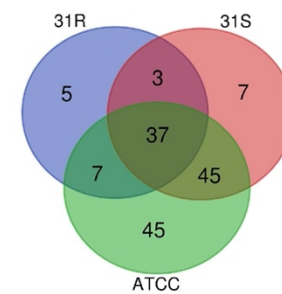

B

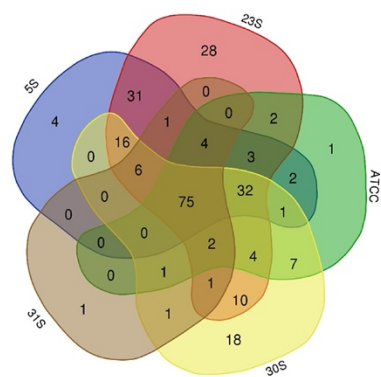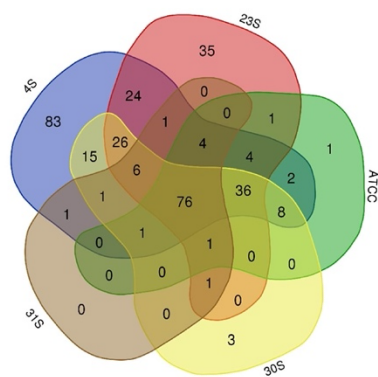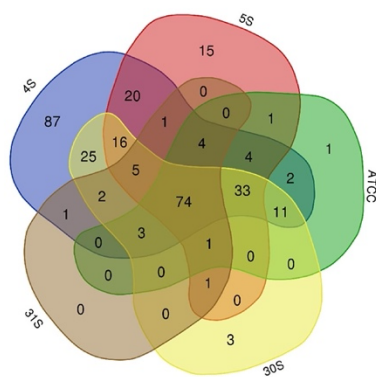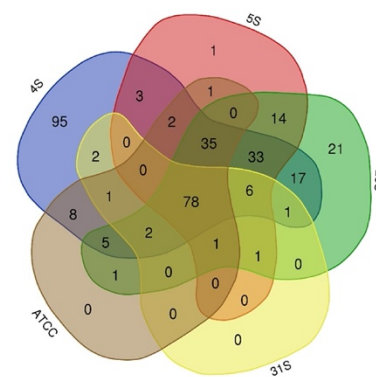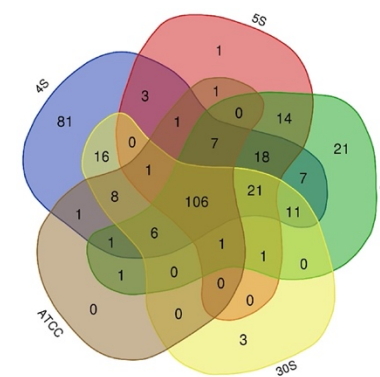

Supplement: FIG S2 [file msystems.00254-22-sf002.pdf]

A

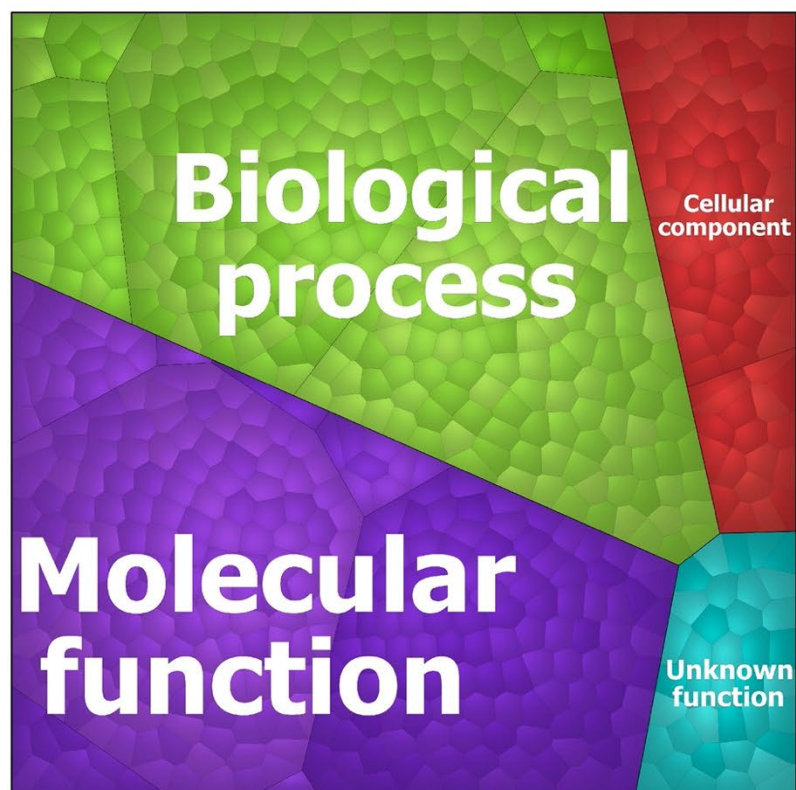

B

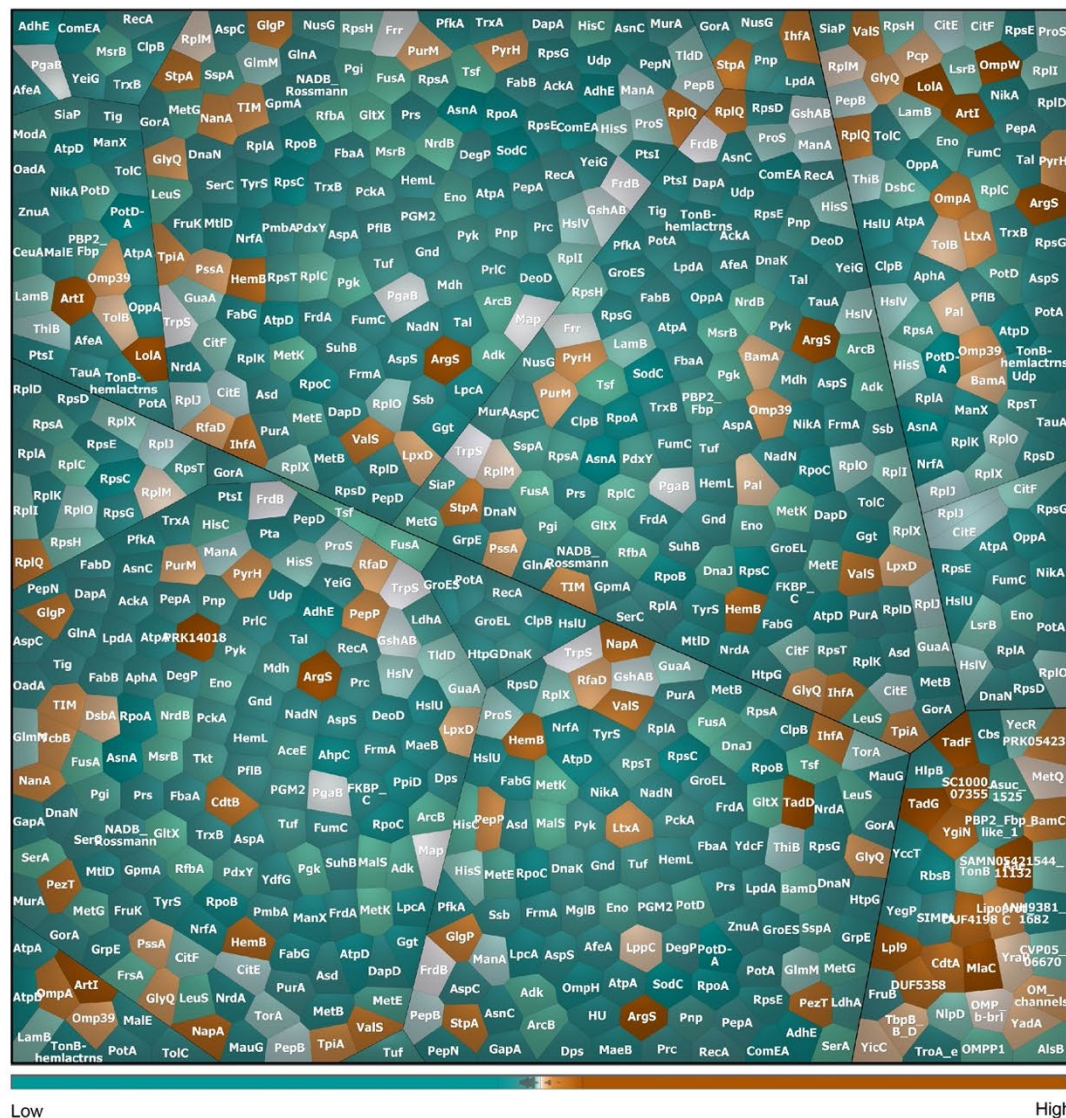

Supplement: FIG S3 [file msystems.00254-22-sf003.pdf]
