# Supplementary material for: Connections between Exoproteome Heterogeneity and Virulence in the Oral Pathogen Aggregatibacter actinomycetemcomitans
Source: mSystems. 2022 Jun 13;7(3):e00254-22. doi: 10.1128/msystems.00254-22 (PMC9239275; doi:10.1128/msystems.00254-22)
Supplement: TABLE S1 [file msystems.00254-22-st001.pdf]

Supplementary Table S1. Genetic variations in different *Aa* pairs

Pairs	Position*	Mutation	Annotation	Description	Gene
4R VS 4S	861599	Δ1bp	Intergenic	Flp pilus assembly protein, pilin Flp/Metal-dependent hydrolase YbeY, involved in rRNA and /or ribosome maturation and assembly	*flp* promotor
5R VS 5S	863134	T→C	Intergenic	Flp pilus assembly protein, pilin Flp /Phosphate starvation-inducible protein PhoH, predicted ATPase	*flp* promotor
23R VS 23S	863092	T→C	Intergenic	Flp pilus assembly protein, pilin Flp /Phosphate starvation-inducible protein PhoH, predicted ATPase	*flp* promotor
30R VS 30S	894165	T→G	Intergenic	Flp pilus assembly protein, pilin Flp /Phosphate starvation-inducible protein PhoH, predicted ATPase	*flp* promotor
31R VS 31S	1029359	T→A	Genic	Single-stranded-DNA-specific exonuclease RecJ	*recJ*
4R VS ATCC	1478055	Δ1bp	Intergenic	Metal-dependent hydrolase YbeY, involved in rRNA and /or ribosome maturation and assembly/ Flp pilus assembly protein, pilin Flp	*flp* promotor
5R VS ATCC	863123	Δ1bp	Intergenic	Flp pilus assembly protein, pilin Flp /Phosphate starvation-inducible protein PhoH, predicted ATPase	*flp* promotor
23R VS ATCC	1539574	Δ1bp	Intergenic	Flp pilus assembly protein, pilin Flp /Phosphate starvation-inducible protein PhoH, predicted ATPase	*flp* promotor
30R VS ATCC	894167	Δ1bp	Intergenic	Flp pilus assembly protein, pilin Flp /Phosphate starvation-inducible protein PhoH, predicted ATPase	*flp* promotor
31R VS ATCC	884999	Δ1bp	Intergenic	Flp pilus assembly protein, pilin Flp /Phosphate starvation-inducible protein PhoH, predicted ATPase	*flp* promotor

*Please note that the position of the SNPs in the pairwise comparisons of R isolates and their derivative S strains refers to the position in the genome sequences of the S strains submitted to NCBI. The position of the SNP detected in the *flp* promoter region of the ATCC 29522 type strain of *Aa* refers to the respective position in the genome of the R isolate that was used in the respective SNPs analysis.
